# Biological function of sialic acid and sialylation in human health and disease

**DOI:** 10.1038/s41420-024-02180-3

**Published:** 2024-09-30

**Authors:** Wengen Zhu, Yue Zhou, Linjuan Guo, Shenghui Feng

**Affiliations:** 1 Department of Cardiology, The First Affiliated Hospital of Sun Yat-Sen University, Guangzhou, China; 2 Department of Ophthalmology, The Second Affiliated Hospital of Guangzhou Medical University, Guangzhou, China; 3 Department of Cardiology, Jiangxi Provincial People’s Hospital, The First Affiliated Hospital of Nanchang Medical College, Nanchang, China; 4 Fuwai Hospital, National Center for Cardiovascular Diseases, Chinese Academy of Medical Sciences and Peking Union Medical College, Beijing, China

**Keywords:** Post-translational modifications, Diseases

## Abstract

Sialic acids are predominantly found at the terminal ends of glycoproteins and glycolipids and play key roles in cellular communication and function. The process of sialylation, a form of post-translational modification, involves the covalent attachment of sialic acid to the terminal residues of oligosaccharides and glycoproteins. This modification not only provides a layer of electrostatic repulsion to cells but also serves as a receptor for various biological signaling pathways. Sialylation is involved in several pathophysiological processes. Given its multifaceted involvement in cellular functions, sialylation presents a promising avenue for therapeutic intervention. Current studies are exploring agents that target sialic acid residues on sialoglycans or the sialylation process. These efforts are particularly focused on the fields of cancer therapy, stroke treatment, antiviral strategies, and therapies for central nervous system disorders. In this review, we aimed to summarize the biological functions of sialic acid and the process of sialylation, explore their roles in various pathophysiological contexts, and discuss their potential applications in the development of novel therapeutics.

## FACTS

- Sialic acids are negatively charged, nine-carbon monosaccharides that play pivotal roles in cellular communication and function.
- Sialylation, a novel post-translational modification driven by adding a sialic acid to the terminal residues of oligosaccharides and glycoproteins, takes part in multiple pathophysiological processes.
- Sialylation not only provides a layer of electrostatic repulsion to cells but also serves as a receptor for various biological signaling pathways.
- Several studies have indicated that sialylation may present a promising avenue for therapeutic intervention.

## OPEN QUESTIONS

- How do alterations in the sialylation process impact the development and progression of diseases beyond the currently studied systems?
- What are the underlying molecular mechanisms that link sialylation to neuropsychiatric disorders, and how can these insights be applied to develop novel therapeutic strategies?
- Can sialylation serve as a therapeutic target for diseases (e.g., cancer, stroke, and cardiovascular diseases), and what would be the potential benefits and challenges?

## Introduction

**Fig. 1:**
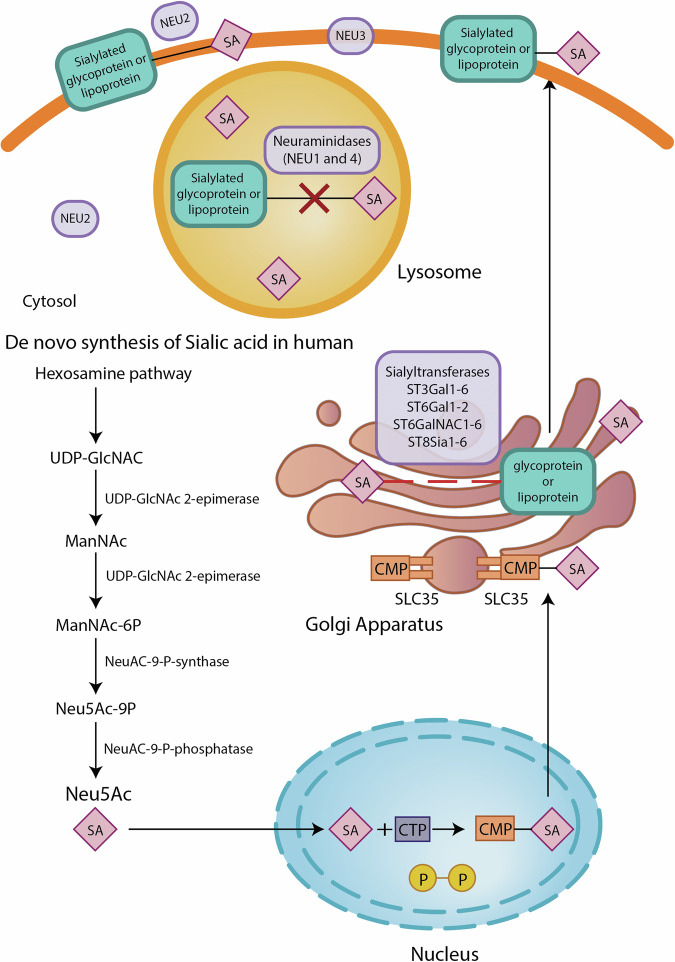
The biological process of sialylation. SA sialic acid, NEU neuraminidases P-P diphosphate, ST3Gals β-galactoside α-2,3-sialyltransferases, ST6Gals β-galactoside α-2,6-Sialyltranferase, ST8Sias α-2,8-sialyltransferases, CTP cytidine-5’-triphosphate, CMP cytdine 5’-monophosphate, SLC 35 Solute Carrier Family 35, Neu5AC N-acetylneuraminic acid.

Sialic acid, a member of the nine-carbon monosaccharides with a keto acid functional group [1], is ubiquitous across vertebrate tissues [2]. First isolated by Blix et al. from submaxillary mucin in 1936, it was named “sialic acid” due to its acidic nature and origin from saliva [3]. To date, over 50 distinct sialic acid species have been identified, including N-acetylneuraminic acid (Neu5Ac), N-glycolylneuraminic acid (Neu5Gc), deaminoneuraminic acid (Kdn), and their modified derivatives such as methylation, acetylation, and sulfation at various positions [4]. Among these, Neu5Ac and Neu5Gc are the predominant forms in mammals. In humans, only Neu5Ac is synthesized de novo, as a mutation in the gene of cytidine monophosphate-N-acetylneuraminic acid hydroxylase has rendered humans unable to convert Neu5Ac into Neu5Gc [5] (Fig. 1). However, Neu5Gc can still be found in certain human cells, especially endothelial and epithelial cells, due to dietary intake [6]. The presence of anti-Neu5Gc antibodies in the human body suggests that antigen-antibody interactions involving Neu5Gc may contribute to chronic inflammation and the increased incidence of diet-related carcinomas and other diseases [7]. Moreover, sialic acid-containing structures are integral to numerous physiological and pathological processes through carbohydrate-protein interactions.

Sialylation, the process of appending sialic acid units to the terminal of lipoproteins and glycoproteins, is a novel form of post-translational modification (PTM) [8], making sialic acids as the “bridging” molecules between cells and their extracellular matrix [4]. In mammals, terminal sialic acids are presented either as single entities or as polysialic acid (PolySia) chains on N- and O-linked glycans of glycoproteins and glycolipids. This process occurs through α-2,3- or α-2,6-bonds to galactose (Gal) or N-acetylgalactosamine (GalNAc) units of glycans, or through α-2,8- or α-2,9-bonds to other sialic acid moieties [4, 9] (Table 1). The dynamic addition and removal of sialic acid serve to regulate structural stability and cell recognition and communication [10–12]. For instance, increased terminal sialylation enhances the serum half-life of glycoproteins such as tissue plasminogen activator (tPA) and erythropoietin (EPO) [13–16]. In addition, sialylation of N-glycans prevents their interaction with the asialoglycoprotein receptor, thereby avoiding liver clearance [17].

**Table 1:** A simplified overview of major sialylation patterns.

Sialylation pattern	Mediated enzyme	Ligation patterns	Representative sialylated molecules
α-2,3 linked	ST3Gals		Sialyl-Lewis X, Sialyl-Lewis A
α-2,6 linked	ST6Gals		Sialyl Tn, APP, VWF
α-2,8 linked	ST8Sias		NCAM, CCR7, CD36, ESL-1, Megalin, NRP2, ST8Sia2, ST8Sia3, ST8Sia4, SynCAM1
			Sialic acids joined internally by α-2,4, α-2,5 O-glycolyl α-2,8, α-2,9, and α-2,8/9 linkages; Polymeric elongation appears at position C8 of a2,3- or a2,6-linked Sia. The value of n varies from 2 to 400.

*ST3Gals* β-galactoside α-2,3-sialyltransferases, *ST6Gals* β-galactoside α-2,6-Sialyltranferase, *ST8Sias* α-2,8-sialyltransferases, *PolySia* polysialic acid, *Gal* galactose, *GalNAc* N-acetylgalactosamine, *APP* Amyloid precursor protein, *VWF* Von Willebrand factor, *NCAM* neural cell adhesion molecule, *CCR7* C-C chemokine receptor type 7, *ESL-1* E-selectin ligand-1, *NRP2* Neuropilin-2, *SynCAM1* Synaptic Cell Adhesion Molecule 1.

In this review, we summarized the current literature on sialic acid metabolism and its impact on various pathophysiological processes. We further explored the therapeutic potential of targeting terminal sialic acids on sialoglycans in disease conditions. Moreover, we highlighted several unresolved questions regarding the effects of aberrant sialic acid metabolism on cellular activities.

## Biological functions of sialic acids

### Sialic acids as anti-adhesive molecules on the cell surface

Sialic acid is recognized as an anti-adhesive glycotype, significantly influencing the biophysical properties of sialylated cells. A prime example is the erythrocyte, which is heavily sialylated and negatively charged [18]. The vascular endothelium’s luminal surface is similarly rich in sialic acid residues [19], creating a charge repulsion that prevents erythrocyte adhesion and facilitates their unimpeded transit through the circulatory system freely. In the study by Weber et al., the varying degrees of physiologic sialylation on intercellular adhesion molecule-2, highly expressed on platelets and endothelium and a counter-receptor for leukocyte integrins and lymphocyte function-associated antigen-1, could influence endothelial and platelet adhesion behaviors [20].

### Sialic acids on receptors

Sialic acids have key roles in intercellular signaling through specific ligands: sialic acid-binding immunoglobulin-like lectins (SIGLECs) and selectins. The interaction between these molecules is facilitated by a salt bridge between arginine and the carboxyl group of Neu5Ac [21]. Upon sialic acid binding, SIGLECs could interact with the DNAX activation protein 12 (DAP12) [22]. Selectins, crucial for leukocyte trafficking to secondary lymphoid organs and infection sites [23], recognize the sialyl-Lewis X (sLex) ligand, which is essential for their interaction with glycoproteins and glycolipids. sLex formation involves the sequential addition of a sialic acid in α-2,3 linkage to a lactosamine unit’s galactose residue, followed by the attachment of a fucose residue in α-1,3 linkage to the N-acetyl-glucosamine unit [23].

## Biosynthesis pathway of sialylation

Sialic acid metabolism is regulated by sialyltransferases and sialidases. Sialyltransferases, located on the type II membrane protein of the Golgi apparatus, catalyze the transfer of sialic acid from a glycosyl donor CMP-Neu5Ac to the terminal positions of oligosaccharides and glycoconjugates. Sialyltransferases are categorized based on the position of sialic acid addition: β-galactoside α-2,3-sialyltransferases (ST3Gals), β-galactoside α-2,6-Sialyltransferases (ST6Gals), and α-2,8-sialyltransferases (ST8Sia). Sialidases, or neuraminidases (NEU), mediate the desialylation process and are classified into four types: NEU1, NEU2, NEU3, and NEU4. NEU1, primarily lysosomal, is involved in exocytosis, immune response, phagocytosis, and elastic fiber assembly. NEU2, predominantly found in the cytosol and plasma membrane, participates in myoblast and neuronal differentiation. Both NEU3 and NEU4 are implicated in neuronal differentiation, apoptosis, and adhesion, with NEU3 localized to the plasma membrane and NEU4 found in lysosomes, mitochondria, and the endoplasmic reticulum [24, 25].

The sialylation-modified cell complex structures, or sialome, are recognized by various sialic acid-binding proteins, initiating multiple sialic acid-dependent signaling pathways [26]. Sialylation is also integral to several physiological functions such as protein conformation regulation, cell proliferation, migration, apoptosis, and cognitive processes [27, 28].

## Sialylation in physiological processes

### Sialylation in the immune system

Sialylation plays a multifaceted role in the immune system. It participates in immune responses and leukocyte trafficking through interactions with SIGLECs and selectins. In addition, sialylation of the Fc fragment of antibodies could modulate the function of antibody [29].

#### Regulation of complement activation by sialic acids

Sialic acids modulate the alternative pathway of complement activation [30]. Factor H, a key mediator in this process, recognizes sialic acids as “self”, facilitating their recruitment on the surface of native cells and downregulating the continuous activation of the complement pathway [31]. This mechanism is characterized by the accelerated dissociation of the C3bBb convertase and the promotion of factor I-mediated C3b cleavage [32]. The type of glycosidic linkage of sialic acid to the glycan structure can influence this recognition [33, 34], and alterations in sialic acid side-chain O-acetylation can also affect factor H binding [35]. Notably, the binding of O-acetylated sialic acids on murine erythrocytes may limit the control of alternative complement pathway activation, as these modified forms are less effective targets for factor H binding [36]. Ficolins, soluble activators of the complement lectin pathway, also recognize sialic acids, particularly on sialylated bacteria, representing a host response to molecular mimicry [37–39].

#### Sialic acids and their ligands in the immune system

**Fig. 2:**
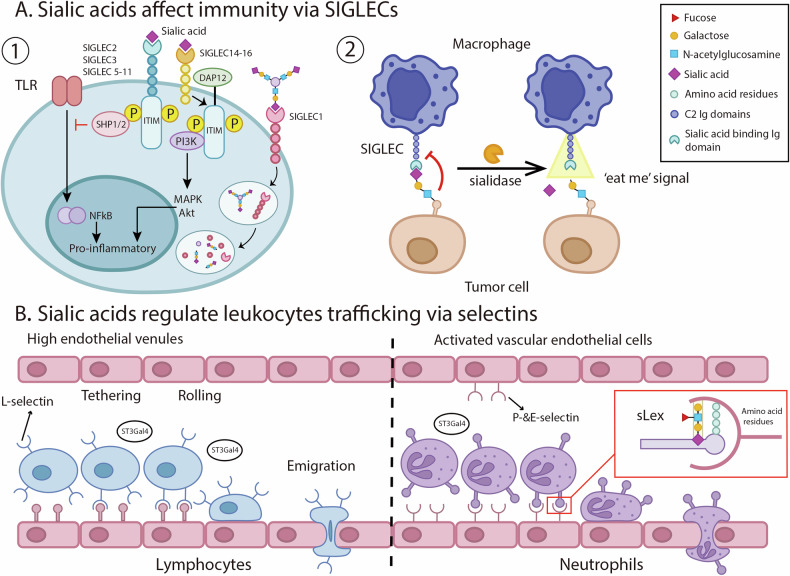
Regulatory roles of sialic acids in the immune system. **A** Modulation of Immune Responses by SIGLECs; **B** Leukocyte Trafficking Facilitated by Sialic Acid. SIGLECs sialic acid-binding immunoglobulin-like lectins, SHP Small Heterodimer Partner, ITIM immunoreceptor tyrosine-based inhibition motif, TLR toll-like receptor, DAP12 DNAX activation protein 12, SLex sialyl-Lewis X, PI3K phosphoinositide-3 kinase.

SIGLECs, a family of sialic acid-binding proteins predominantly found in immune cells such as leukocytes and macrophages, are known to regulate inflammatory signals [31, 40, 41]. In sepsis models, SIGLEC-G and SIGLEC-E stimulation has demonstrated significant anti-inflammatory potential, suggesting the therapeutic value of SIGLEC-targeting treatments [42–44]. SIGLEC agonists also modulate adaptive immune responses, for example, SIGLEC-G binding to CD24 on antigen-presenting cells can mitigate T-cell-mediated responses in graft-versus-host disease models [45]. In humans, SIGLECs are categorized into two groups: SIGLEC-1 (Sialoadhesin), SIGLEC-2 (CD22), SSIGLEC-4 (MAG), SIGLEC-15 [46], and the CD33-related SIGLECs (SIGLEC-3, SIGLEC 5-11, SIGLEC-14, and SIGLEC-16) [47]. Sialylation of SIGLEC-1 can induce internalization of SIGLECs and antigens, initiating immune responses by presenting antigens to dendritic cells (DCs) or B cells. Conversely, sialylation of SIGLEC-2, 3, and 5-11 can suppress proinflammatory signals by modulating toll-like receptor signaling, while SIGLEC-14, -15, and -16 activation by sialic acids can stimulate proinflammatory responses via the MAPK and AKT signaling pathways [46] (Fig. 2). In tumor cells, the removal of sialic acid residues from glycans leads to the exposure of galactose residues, which in turn generate “eat me” signals recognized by both professional and non-professional phagocytes, including microglia [48] (Fig. 2).

Another sialylated ligand, sLex, is found in CD45RO^+^ memory-phenotype subsets of human T cells and is upregulated on CD45RA^+^ naïve human CD4^+^ and CD8^+^ T cells following accepting T cell receptor stimulation [49]. Upon stimulation with cytokines such as IL-2 in combination with IL-12 or IL-15, CD4^+^ and CD8^+^ T cells expressing the sLex antigen in human peripheral blood mononuclear cells are rapidly activated in an antigen-independent manner. Importantly, sLex-positive human CD8+ T cells have been shown to significantly enhance reverse antibody-dependent cellular cytotoxicity (ADCC) compared to sLex-negative cells [49]. These observations suggest that sLex-expressing memory CD4^+^ and CD8^+^ T cells contribute to early-stage immunity through the provision of IFN-γ and cytotoxicity.

#### Leukocyte trafficking and selectin ligands

Selectin ligands play crucial roles in modulating leukocyte trafficking, a process vital for immune defense and surveillance. Leukocyte migration across body compartments involves capture, rolling along blood vessels, firm arrest on the endothelial lining, and leukocyte modification [50] (Fig. 2). The initial steps of capture and rolling are mediated by interactions between selectins and α-2,3-sialylated carbohydrate determinants on selectin ligands. ST3Gal5 has been shown to influence chemokine-triggered leukocyte arrest by expanding the role of α-2,3 sialylation in leukocyte rolling and subsequent arrest [51]. Sialylation by ST3Gal4 is also essential for leukocyte trafficking, with ST3Gal4^–/–^ mice exhibiting reduced adhesion during inflammation. Collectively, the evidence underscores the importance of sialylation of selectin ligands on chemokine receptors in maintaining the integrity of leukocyte trafficking processes.

#### Sialylation of antibody Fc fragments

Sialic acid binding to the Fc fragment of antibodies significantly modulates their effector functions [52]. Human IgG contains predominantly α-2,6-linked sialic acid residues [53, 54], whereas recombinant IgGs with α-2,3-sialylation have been successfully expressed in Chinese Hamster Ovary cells. Mouse myeloma cells are capable of producing recombinant IgGs with both α-2,3-linked and α-2,6-linked sialic acid residues, without raising concerns regarding immunogenicity [17]. Elevated Fc sialylation has been demonstrated to reduce the ADCC activity, potentially due to suppressed binding affinity [52].

Prior studies have found that intravenous IgG fractions containing α-2,6-linked sialic acid residues in the Fc region exhibit enhanced anti-inflammatory properties [55, 56]. It may be explained that this involves the binding of Fc-sialylated IgG to mouse SIGNR1 or its human counterpart, DC-SIGN, which may contribute to the anti-inflammatory effects by upregulating the inhibitory Fc receptor FcgRIIB on macrophages and DCs [57, 58]. However, this hypothesis has been subject to recent scrutiny, with other factors also being considered. For instance, sialylated glycans constitute about 15% of total N-glycans in intravenous IgG, while Fab glycans, which can be more complex and carry both α-2,3-linked and α-2,6-linked sialic acid residues, account for 15–30%. The contribution of Fab glycans with different sialylation patterns to the anti-inflammatory effects of intravenous IgG is not yet clear but may be significant. In addition, the reduced cell-killing ability of Fc-sialylated antibodies could also contribute to their anti-inflammatory properties, although the exact mechanisms require further investigation. Furthermore, sialylation of IgG can decrease its binding affinity to insoluble or cell surface antigens, an effect not observed with soluble antigens [52]. The negative charge of sialic acid residues may alter the overall charge of the antibody molecule, lowering its isoelectric point and affecting antigen binding.

The sialylation of the IgG Fc fragment has also been linked to negative effects. Following immune checkpoint blockade therapy for hepatocellular carcinoma, effector T cells can induce IgG sialylation through an IFN-γ-ST6Gal-I-dependent pathway [59]. Sialylated IgG primarily targets DC-SIGN macrophages, which, upon stimulation, can elevate ATF3 through Raf-1, inhibiting the cGAS-STING pathway and suppressing type-I-IFN-mediated antitumor immunity [59].

#### Sialylation and protease sensitivity

Sialylation increases the sensitivity of antibodies to proteases, potentially due to the negative charge of sialic acid residues. Since many proteases are acidic, sialylated antibodies may reduce the effective pI of these enzymes [17]. Structural changes in the CH2 domains of antibodies due to sialylation could also enhance sensitivity. The limited space in the CH2 domain may be affected by the bulkier sialic acid residues, leading to structural and functional alterations. The addition of sialic acid could cause a bulge in the Fc fragment, increasing amino acid flexibility and improving the accessibility of antibodies to proteases.

#### Sialylation and dendritic cells

Sialylation impacts the maturation and function of DCs [60]. Sialidase treatment of DCs and sialyltransferase knock-out mice models have shown increased DC maturation [60–63]. In the absence of sialylation, human monocyte-derived DCs exhibit increased bacterial phagocytosis [61–63]. Polysialylation of CCR7 [64] in mature DCs is essential for CCL21 ligand recognition and chemokine-mediated migration. In addition, polysialylation of neural cell adhesion molecule (NCAM) in natural killer (NK) cells influences the fate of DCs, with polysialylated NK cells binding to DC-SIGN ligands and protecting SIGN-expressing DCs from NCAM-positive NK cell-mediated cytotoxicity [65].

### Sialylation and stem cell pluripotency

Sialylation is a critical process for establishing and maintaining stem cell pluripotency. Undifferentiated human induced pluripotent stem cells (iPSCs) exhibit higher levels of ST6Gal1 expression compared to non-pluripotent cells [66]. Knockdown of the ST6Gal1 gene or the use of sialyltransferase inhibitors can negatively impact the efficiency of somatic cell reprogramming. Several cell adhesion molecules, including E-cadherin, integrins, and catenins, are sialylated glycoproteins [67]. Aberrant sialylation can disrupt the interactions between these adhesion molecules and their receptors, blocking signal transduction related to cell differentiation and thus preserving stem cell characteristics.

### Sialylation in sperm development and fertilization

Sperm exhibit millions of sialylation sites [68], and the levels of sialylation and sialidase activity undergo dynamic changes during the processes of sperm maturation, capacitation, and sperm-egg binding [69, 70], which are closely related to successful fertilization and embryonic development [8, 71].

Sperm are anatomically divided into the head, neck, and tail regions, with different types of SIGLECs. These proteins are significantly expressed in the principal piece of the sperm, facilitating tail-specific functions through sialylation-mediated intracellular communication [72]. Since sperm flagellar proteins are glycoproteins rich in sialylation [73], treatment with sialidase significantly reduces the activity of their forward motility proteins [74]. In the human testis, the sialic acid metabolism pathway is more prevalent in Sertoli cells compared to other testicular cells. Sialyltransferases, including ST3Gal1 and ST6 GalNAC6, are expressed at higher levels in male reproductive tissues [75], indicating the vital role of sialylation in male fertility.

Glycoproteins with high sialylation levels, such as beta-defensin 126 (DEFB126), are necessary for the smooth entry of sperm into the female reproductive tract to meet the eggs [76, 77]. The specific roles of sialylation include: creating a negatively charged surface on the sperm, reducing resistance in the negatively charged cervical mucus; interacting with SIGLECs and other recognition molecules on the epithelium of the fallopian tube and immune cells, preventing sperm from being attacked in the female reproductive tract, and maintaining temporary storage in the fallopian tubes; and during sperm capacitation in the female reproductive tract, a gradual decrease in the degree of sialylation on the sperm surface occurs. Ultimately, sperm recognize the zona pellucida receptor of the egg through their sialylation, initiating the acrosome reaction and facilitating fertilization [75]. In addition, PolySia is suggested to potentially regulate sperm development by affecting the communication between germ cells and Sertoli cells [78]. Polysialic acid can bind various growth and nutritional factors [79], which may benefit the survival of male reproductive cells [80]. Furthermore, polysialylated-neural cell adhesion molecule (PSA-NCAM) is widely expressed in mammalian testes, epididymal smooth muscle cell clusters, and mature human sperm [75, 81], although the exact mechanisms remain unclear [82].

### Sialylation in maintaining platelet function

A case of thrombocytopenia with macrothrombocytopaenia has been documented in a 70-year-old patient with a deficiency in sialylation ability [83]. Despite normal platelet precursor and megakaryocyte counts and morphology, the increased platelet size suggests cellular fragmentation defects. Similar findings are observed in Bernard-Soulier syndrome, a macrothrombocytopenia caused by a deficiency in the major sialic acid carrier, platelet surface glycoprotein Ib [23, 84, 85]. These indicate a potential role for sialylation in platelet disorders.

Von Willebrand factor (VWF), a crucial molecule in the coagulation system, undergoes complex PTMs before being secreted into plasma. VWF monomers, which contain numerous N-glycan and O-glycan structures, are capped with terminal sialic acid residues that impart a negative charge [86]. Notably, the sialylation status of VWF is heterogeneous and dynamic; VWF from endothelial cells is fully sialylated, whereas this level is significantly lower in VWF from platelets. This difference may be due to varying activities of their sialyltransferases in cells.

Sialylation significantly influences VWF function in multiple ways, including its activity, susceptibility to proteolysis, and clearance [86]. Federici et al. demonstrated that treatment of VWF with neuraminidase can remove over 95% of the total sialic acid content [87]. When using inhibitors to suppress the function of this enzyme’s activity, desialylation did not have directly influence on the multimer pattern [88]. However, in platelet-rich plasma, desialylated VWF can induce spontaneous platelet aggregation and effectively modulate platelet adhesion to collagen under shear conditions [87].

## Sialylation in pathological processes

### Roles of sialylation in cancer

#### Cancer progression

**Fig. 3:**
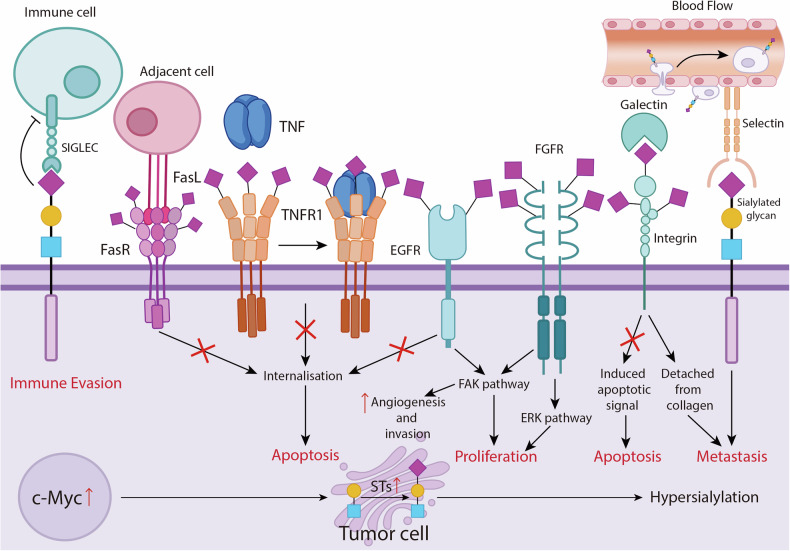
Impact of Sialylation on Cancer Progression. SIGLECs sialic acid-binding immunoglobulin-like lectins, FasR Fas receptor, TNFR tumor necrosis factor receptor, FGFR fibroblast growth-factor receptor, ERK extracellular signal-regulated kinase, FAK focal adhesion kinase, EGFR epidermal growth factor receptor, STs Sulfotransferases.

Hypersialylation, characterized by alterations in sialic acid levels, sialidase activity, sialyltransferase activity, or sialoproteins [89], has been observed in most tumor cells. This elevated sialylation level enhances tumor cell resistance to apoptosis and promotes proliferation. Tumor cells often upregulate ST6GAL1, leading to increased α2-6-sialylation of the Fas receptor (FasR) and tumor necrosis factor receptor 1, which suppresses cancer apoptosis and facilitates the formation of secondary tumor sites [90]. In addition, sialylation of integrins can block apoptotic signals, preventing tumor cell death [91] (Fig. 3). In various malignancies, elevated levels of serum or plasma total sialic acid are detectable during tumor initiation, progression, and treatment [92–95]. The hypersialylation state of tumor cells may accelerate cancer progression through mechanisms such as cell-cell repulsion, altered binding to the extracellular matrix, enhanced migration, and invasion, all of which contribute to metastasis formation and poor disease prognosis.

#### Cancer metastasis

Metastasis can be summarized in four steps: escaping from the primary site, survival in the bloodstream, lymphatic transfer, and attachment to new distal sites [96]. Sialylation plays a vital role in each of these processes. For instance, α2-6-sialylation of the epidermal growth factor receptor (EGFR) has been shown to regulate the epithelial-mesenchymal transition (EMT) of cancer cells [97], influence membrane retention, regulate integrin tension, and affect focal adhesion and cell motility [98, 99]. Increased α2-6-sialylation of β1 integrin enhances its binding to collagen I, promoting tumor migration and invasion [100]. Similarly, α2-6-sialylation of the fibroblast growth factor receptor (FGFR) amplifies signals through extracellular regulated protein kinases 1/2 (ERK1/2) and focal adhesion kinase (FAK) [101]. α2,3-Sialylated CD44 promotes adhesion to hyaluronic acid [102], further accelerating cancer cell motility and metastasis. Poor prognosis in patients is often associated with the sialylation of tumor-associated carbohydrate antigens like Sialyl Tn (STn), which can enhance cancer invasiveness [103, 104]. Under hypoxic conditions, PolySia maintains tumor cell migration [105], as the hypoxic microenvironment promotes the polysialylation of NCAM, increasing the motility of glioblastoma cells [106] (Fig. 3).

However, the role of sialylation in cancer metastasis may be complex and different among various malignancies. In estrogen receptor-positive breast cancers, high levels of ST6GalNAc2 increase the sialylation of core 1 antigen, reducing the binding of galectin-3 and thus tumor metastasis. In contrast, estrogen receptor-negative breast cancers with low ST6GalNAc2 exhibit higher endothelial cell adhesion and metastasis [107]. In addition, sialic acid-containing GM3 can reduce phosphoinositide-3 kinase (PI3K)-AKT signaling, increasing the migration and invasion of breast and colon cancer cells [108].

#### Immune evasion

Sialic acids play a crucial role in cell-environment interactions and are integral to self-recognition through self-associated molecular patterns. An upregulation of sialoglycans on the surface of malignancies creates an “antigenic masking” effect, significantly influencing tumor immunogenicity and enabling the concealment of tumor-associated antigens [109]. The dense layer of sialoglycans on the tumor cell surface generates steric and electrostatic barriers, effectively masking underlying glycans and protein epitopes to evade immune cell recognition [64].

During tumor progression, sialic acid-binding antigens can act as “don’t eat me” signals that interfere with macrophage function [110, 111], and these signals can also be transmitted to NK cells and T cells, inhibiting their activity. Heavy glycosylation of tumor-derived MUC1 can prevent the degradation of sialic acids in endosomes, thereby creating a barrier to antigen presentation by DCs [112].

### Sialylation in neurological disorders

The concentration of sialic acid is highest in mammalian central nervous system (CNS), with 65% in gangliosides, 32% in glycoproteins, and 3% free sialic acid [113]. Most sialic acids are incorporated into gangliosides, while polySia is linked to glycoproteins such as NCAM in the CNS [44]. The expression of PolySia is predominantly detected in four types of cells including migrating neuroblast cells (e.g., olfactory neuronal precursors), extending cells (neurons and Schwann cells), synapses in synaptic plasticity regions, and neural stem cells (subventricular zone) [44, 114]. In brain, the rapid changes of sialylation in cell-surface may occur physiologically, usually induced by the transfer of NEU1 or NEU4 to the cell surface [115]. Acute stress could induce the rapid decrease of polysialylation in olfactory bulb and prefrontal cortex in mice, which is mediated by sialidases from microglia and astrocytes [116]. Moreover, neural activity instantly increases the activity of sialidase activity on neuronal and astrocytic surface, causing neuronal desialylation, which in turn modifies memory formation [117].

#### Alzheimer’s disease

Alzheimer’s disease is characterized by the deposition of Aβ peptides in the brain, which is central to the “amyloid cascade hypothesis” of the disease’s development [118]. Sialylation in the Golgi apparatus modulates the functions of the amyloid precursor protein (APP) [28]. Overexpression of ST6Gal-I in Neuro2a cells enhances α-2,6-sialylation of APP, increasing the extracellular levels of Aβ peptides, sAPPβ, and sAPPα. Conversely, Aβ peptide secretion is significantly reduced in cells lacking ST6Gal-I [119]. The N-glycans on APP are essential for this enhanced secretion, and a correlation between α-2,6-sialylated APP and sAPPβ levels has been observed in the mouse brain [119].

Genetic variants of SIGLEC genes may also contribute to Alzheimer’s disease by affecting microglial cell functions. Microglia express SIGLEC-3/CD33, and its polymorphisms are associated with varying risks of Alzheimer’s disease. The inhibitory SIGLEC receptors, mediated by TYRO protein tyrosine kinase-binding protein (TYROBP), can suppress phagocytosis and promote oxidative burst, inflammation, migration, and proliferation [44]. In addition, loss-of-function mutations in triggering receptor expressed on myeloid cells 2, which is upregulated by CD33-related SIGLEC receptors, are linked to a nonresponsive microglial phenotype and an increased risk of Alzheimer’s disease [120].

#### Multiple sclerosis

Multiple sclerosis is an autoimmune disease involving the CNS, where B cells play a significant role [121]. CD22 and SIGLEC-G/SIGLEC-10 on B cells are crucial for maintaining B cell tolerance, and their deficiency can lead to autoimmunity. Antigen-specific B cell tolerance may be induced by targeting SIGLECs on the B cell receptor or antigen complex. SIGLEC-2/CD22 or SIGLEC-G-displaying nanoparticles can suppress antigen-specific B cell activation, leading to B cell apoptosis [122–124].

#### Acute nervous system injury

Local overexpression of PolySia can promote axonal regrowth and neural connectivity [125–128]. Similarly, PolySia-expressing Schwann cells enhance Purkinje axonal regeneration post-injury [126]. However, long-term overexpression of PolySia may impede myelination or remyelination [125, 129–131], as it acts as a ‘repulsive strut’ on unmyelinated or demyelinated axons. PolySia-mimicking peptides, such as tegaserod or 5-nonyloxytryptamine oxalate, have been developed to foster repair in the spinal cord [132, 133] and peripheral nerve injuries [134–136]. This strategy has been recently confirmed by a collagen–laminin scaffold-based tissue engineering approach [137].

#### Prion diseases

Prion diseases are transmissible neurodegenerative disorders caused by misfolded sialoglycoprotein, the prion protein (PrPC) [138, 139]. PrPC undergoes PTMs, including the addition of sialic acids to N-linked glycans and a glycosylphosphatidylinositol (GPI) anchor [140, 141]. Sialic acids are linked to the termini of these N-linked glycans through α2-3 or α2-6 linkages, and variations in N-linked glycan structure result in numerous PrPC glycoforms [141, 142]. Recent studies have shown that PrPC molecules with unsialylated GPIs are refractory to conversion into PrPSc [143]. Experiments involving the injection of partially desialylated PrPSc, which exposes more galactose, have demonstrated an inability to induce prion disease in animal models [144–146]. Notably, animals infected with partially desialylated PrPSc were found to be free of prions in their brains [145, 146].

In PrPSc particles, glycans are oriented outward, with terminal sialic acid residues contributing to the negative charge on the PrPSc surface [147–149]. This negative charge leads to electrostatic repulsion between sialic acid residues, imposing structural constraints on PrPSc replication [150]. A portion of heavily sialylated PrPC is less likely to be converted into PrPSc, with the degree of exclusion being strain-specific. Treatment of PrPC with sialidases, which remove these structural constraints, can increase the replication rate of PrPSc in PMCAb cells, with the extent of increase varying widely between different strains [144, 150]. Furthermore, PrPSc-induced toxicity is influenced by PrPC molecules with sialylated GPI anchors [151, 152]. Aggregation of PrPC with sialylated GPIs can cause injury to synapses in vitro by activating cytoplasmic phospholipase A2 in cultured neurons [151, 152]. However, the precise relationship between the sialylation status of N-linked glycans on PrPSc and its toxicity is not fully understood [138, 153, 154].

### Sialylation in cardiovascular diseases

In the context of atherosclerosis, mouse model experiments have shown that the administration of Neu5Ac can reduce atherosclerosis in hyperlipidemic mice [155–157]. Treatment with Neu5Ac in Apoe^−/−^ mice decreased atherosclerotic plaque formation and hepatic lipid accumulation, associated with upregulation of hepatic proteins involved in reverse cholesterol transport and downregulation of inflammatory markers [157]. ApoB, a major component of very-low-density lipoprotein and low-density lipoprotein (LDL), contains sialylated glycosylation sites that are typically modified with complex N-linked glycans terminated with sialic acid [158]. Patients with CVDs have elevated plasma LDL levels with reduced sialic acid content [159, 160]. These hyposialylated LDL particles are more readily taken up by cells from the human aorta, leading to increased intracellular cholesterol ester accumulation [161–163]. Compared to normal LDL, hyposialylated LDL is more immunogenic, promoting the production of proatherogenic autoantibodies [164–167] and accelerating atherosclerosis.

In mouse models, the manipulation of neuraminidase function affects the extent of atherosclerosis. NEU1, a crucial component of the atherogenic action of elastin-derived peptide, stimulates monocyte migration, reactive oxygen species production, LDL oxidation, and vascular smooth muscle cell proliferation [168–170]. Atherosclerotic lesions are significantly reduced in Apoe^−/−^ mice with mutated NEU1 compared to those with only Apoe knockout [171], with fewer macrophages, T cells, and smooth muscle cells indicating reduced inflammation and cell recruitment in the plaque. Suppression of NEU1 also decreases left ventricular dysfunction following ischemia/reperfusion injury [172]. Demina et al. [173] confirmed that the deficiency of NEU1 and NEU3, but not NEU4, attenuates atherosclerosis in mouse models. In addition, increased plasma neuraminidase activity is observed in patients with myocardial infarction [174].

In patients with atheroma or atherosclerosis, sialyltransferase activity is upregulated compared to healthy individuals [175]. In Apoe^−/−^ mice, the deficiency of ST3Gal4 reduces atherosclerotic plaque size and macrophage numbers without affecting plasma cholesterol levels [176]. A genome-wide association study has revealed that single nucleotide polymorphisms in ST6Gal1 are associated with multiple inflammatory disorders, including CVDs [177]. The expression level of ST6Gal1 in the aortic endothelium is inversely related to atheroma formation in Apoe^−/−^ mice [178]. This evidence suggests that sialyltransferases might be potential targets for the prevention and treatment of atherosclerosis.

### Sialylation in virus infection

Sialylation significantly influences viral binding and replication mechanisms. Viruses, such as the influenza virus, bind to sialylated glycans on host cells via hemagglutinin (HA), a process that facilitates membrane fusion and endocytosis. This interaction is mediated by a highly conserved receptor-binding domain on HA, which specifically recognizes α-2,6 linked sialic acids present in human influenza strains [179]. The binding between HA and sialic acid typically occurs through weak hydrophobic and hydrogen bonds, with an affinity measured in the millimolar range for monovalent interactions [180]. However, when multiple HA monomers are involved, the binding affinity increases dramatically, suggesting that multivalent interactions between HA and terminal sialic acid residues are crucial for viral attachment and infection [181–183].

Neuraminidase (NA), another glycoprotein on the influenza virus surface, facilitates the cleavage of HA-sialic acid bonds [180], thereby enhancing viral infectivity. NA cleaves the links between the virus and heavily sialylated mucins in the upper respiratory system [184], which act as decoys. Like HA, NA also preferentially interacts with sialoglycans in α-2,3 or α-2,6 conformations [185].

Hemagglutinin esterase (HE) and hemagglutinin-esterase-fusion protein (HEF) are additional surface glycoproteins that interact with sialic acid. HE is found in coronaviruses, while HEF is present in influenza viruses. These proteins combine the functions of HA and NA, binding to specific 9-O-acetylated sialic acids to enable the release of viral progeny from host cells and evade heavily sialylated decoy cells through receptor destruction activity [186].

### Sialylation in psychiatric disorders

Genome-wide association studies have linked genetic variants of the ST8Sia2 gene, or its loss-of-function mutations, with several major psychiatric conditions, including schizophrenia [139, 187, 188], bipolar disorder [139], and autism [189, 190]. Patients with schizophrenia often exhibit decreased levels of polySia-NCAM [191–193]. Mouse models lacking ST8Sia2 display schizophrenia-like behaviors, such as cognitive dysfunction, impaired prepulse inhibition, and heightened sensitivity to amphetamine-induced locomotion [194]. These findings suggest that structural or functional impairments in polySia may contribute to the development of schizophrenia [188].

## Sialylation in novel therapeutic strategies

### Anti-cancer therapy

Current research suggests that the use of neuraminidase to remove sialic acid from the tumor cell surface can enhance tumor immunogenicity [96]. Therefore, developing drugs that disrupt sialic acid metabolism and its associated signaling pathways is a viable strategy (Supplementary Table).

#### Blockade of sialylation

One approach to anti-cancer therapy involves inhibiting sialyltransferases to block sialylation. Most current sialyltransferase inhibitors are analogs of sialic acids and CMP-sialic acids [195, 196]. A prominent example is 3Fax-Peracetyl Neu5Ac (P-3Fax-Neu5Ac), a sialic acid mimetic that, when modified at the C-5 position, can enhance its potency [197]. P-3Fax-Neu5Ac has been shown to inhibit cancer cell migration and proliferation [198, 199] and, in vivo, to enhance cytotoxic CD8+ T cell-mediated anti-tumor responses [200–202]. In addition, soyasaponin I, another sialyltransferase inhibitor, primarily suppresses cellular ST3Gal activity, reducing tumor cell invasiveness [203]. Other inhibitors, such as 8-keto-sialic acid [204] and endogenous CMP-Neu5Ac [205], are also being investigated for their effects on sialic acid metabolism.

#### Targeting sialic acid’s ligand-receptor interactions

Therapies that target interactions between sialic acid and its receptors, such as selectins and SIGLECs, are under investigation. Uproleselan, which blocks E-selectin, has been shown to limit tumor cell extravasation and adhesion, significantly reducing metastasis [206]. Fucoidan [207], a P-selectin targeted agent, may benefit patients with prostate cancer resistant to docetaxel chemotherapy. Glycopolymers with P-selectin affinity are being explored to prevent melanoma spread [208]. These inhibitors also block E-/P-selectin-mediated signaling in tumor cells, offering additional anti-cancer effects.

As for SIGLECs, its interactions with sialic acid are considered glyco-immune checkpoints for tumor cells [209–212]. Antibodies against SIGLEC-7 and SIGLEC-9 can suppress the conversion of macrophages into tumor-associated macrophages and reprogram the immunosuppressive tumor microenvironment, enhancing anti-cancer immunity [213]. SIGLEC-9 antibodies, in particular, can suppress the inhibitory effects of cytotoxic CD8+ T cells [214, 215], promoting T cell receptor signaling, cytotoxicity, and cytokine production. Antibodies targeting SIGLEC-15 can also enhance anti-tumor immunity and limit cancer progression. The use of SIGLEC-7, SIGLEC-9, and SIGLEC-15 antibodies may provide an alternative therapeutic option for patients resistant to PD-L1/PD-1 blockades [213–217].

#### Conjugating antibodies with sialidase

To overcome the reduced efficacy of PD-L1 antibodies due to glycosylation, an artificial PD-L1 antibody-sialidase conjugate has been developed. This conjugate enhances the blockade efficiency of PD-L1, augmenting anti-cancer activity through T-cell reactivation [218]. Similar effects have been observed with a HER2 antibody-sialidase conjugate, which improves antibody affinity and degrades tumor-derived sialoglycans, boosting the anti-tumor immune response [211]. A corresponding clinical trial (NCT05259696) is investigating the safety, pharmacodynamic effects, and antitumor activity of E-602, a bi-sialidase fusion protein targeting immunosuppressive sialoglycans [219].

#### Sialic acid modification in anti-cancer vaccine development

Sialoglycans, often overexpressed in tumors, are potential targets for novel cancer vaccines. The low immunogenicity of GM3, a sialylated ganglioside expressed by tumor cells, has been addressed by modifying the sialic acid residue [220]. Sialic acid-modified sTn antigenicity has also been shown to elicit high titers of antigen-specific IgG antibodies [221], making Tn-based glycoconjugates promising candidates for vaccination strategies. Cancer-therapeutic vaccines targeting sialic acid are in development, with a synthetic sTn-keyhole limpet hemocyanin vaccine (Theratope®) having completed clinical trials and shown to improve survival in advanced breast cancer [222]. In addition, carbohydrate vaccines, including a pentavalent vaccine (Globo-H-GM2-sTn-TF-Tn) and a heptavalent vaccine (GM2-Globo-H-Lewis Y-Tn-sTn-TF-Tn-MUC1), have successfully elicited IgG and/or IgM responses in ovarian cancer patients [223, 224].

### Stroke treatment

Traditional stroke treatments include thrombolytic therapy with tissue plasminogen activators and surgical clot removal. However, these methods can lead to rapid increases in reactive oxygen species and subsequent ischemia-reperfusion damage [225, 226]. Antioxidants, such as selenium nanoparticles, are being investigated for stroke treatment [227]. Angelica polysaccharide, derived from the Chinese herb Angelica Sinensis, has been studied as a drug delivery system and for its neuroprotective effects against hypoxia-induced apoptosis and autophagy in neural stem cells [228].

Modifying drug delivery systems with sialic acid can improve hydrophilic properties and enhance brain drug delivery efficiency due to the negative charge of the sialylation process [229, 230]. For instance, sialic acid-modified angelica polysaccharide and MSAOR@Cur nanoparticles have shown potential in improving brain infarction and neurological outcomes. The nanoparticle is designed to cross the blood-brain barrier and uses sialic acid-modified angelica polysaccharide as a hydrophilic end, with an oxalate bond linking resveratrol and curcumin to enhance drug delivery [231].

### Respiratory virus infection

Sialylation’s involvement in virus infection has led to the development of drugs targeting interactions between sialic acid and viral surface glycoproteins. Oseltamivir and zanamivir, NA inhibitors, prevent virus spread by inhibiting NA from cleaving bonds between HA and terminal sialic acids on host sialoglycan chains. This action restricts the release of viral progeny from infected cells and prevents the virus from escaping heavily sialylated decoy mucins in the upper respiratory tract [232]. (Supplementary Table).

In addition, inhibitors target to the interactions between HA and sialic acid have also been developed. Peptides that mimic sialic acids, such as Ala-Arg-Leu-Pro-Arg, have been developed to block HA interactions [233]. However, the therapeutic potential of polymerized sialic acid analogs is limited by solubility and cytotoxicity issues [234]. To overcome these challenges, negatively charged liposomes have been used to deliver high-affinity HA-binding sialic acid analogs like sialylneolacto-N-tetraose [235, 236]. These decoy liposomes can suppress viral infectivity by forming multivalent bonds with the HA receptor-binding domain.

DAS-181 (Fludase), a sialidase mimic, is under phase III clinical trials for its potential to block virus infection [237]. It binds to sialylated cell surface polysaccharides [238] and hydrolyzes both α-2,3 and α-2,6 linked terminal sialic acids, demonstrating efficacy against various strains of influenza, parainfluenza, and metapneumoviruses. While DAS-181 has shown relatively few side effects, the development of antibodies against it after long-term use is a concern [239].

Sialyltransferase inhibition represent another antiviral strategy. Inhibiting cell surface sialylation can block virus infection by preventing viral binding to sialoglycans [240]. siRNAs silencing ST6GalI expression have been shown to reduce the infectivity of human influenza strains without affecting cell viability [241]. Existing sialyltransferase inhibitors include a variety of compounds discovered in natural products [242]. However, the antiviral effects of sialyltransferase inhibitors have not been confirmed in in vivo studies [243], indicating a need for further research on sialyltransferase subtype selectivity.

Chloroquine is another small molecule inhibitor of sialic acid expression with broad-spectrum antiviral activity. It inhibits quinone reductase 2, structurally similar to enzymes involved in sialic acid biosynthesis [244, 245]. Chloroquine’s antiviral effects are also associated with the blockade of sialic acid, as it binds to sialic acids and sialoglycans [246], preventing virus attachment.

### Central nervous system disease treatment

SIGLEC-targeting therapies are now being explored for their potential in treating inflammatory CNS injuries, such as multiple sclerosis. B cell-depleting anti-CD20 therapies have shown success in this disease. SIGLEC agonists, particularly CD22 and SIGLEC-G/SIGLEC-10, are crucial for maintaining B cell tolerance, as their deficiency leads to B cell hyperactivation and autoimmune phenotypes in mice. Inducing antigen-specific B cell tolerance by recruiting SIGLECs to the B cell receptor/antigen complex is a promising strategy. Experiments have shown that antigen-bearing polymers or liposomal nanoparticles with synthetic SIGLEC-2/CD22 or SIGLEC-G ligands can significantly suppress the activation of antigen-specific B cells, leading to B cell apoptosis [122, 123, 247]. (Supplementary Table).

In spinal cord injury treatment, preclinical data indicate that local overexpression of polySia promotes axonal regrowth and neural connectivity. Overexpression of polySia or engineered Schwann cells expressing polySia has also been shown to facilitate Purkinje axonal regeneration after brain injury. This suggests that re-expressing polySia in damaged adult CNS tissues may support neural cell remodeling and repair. Research on polySia-mimicking peptides, such as tegaserod and 5-nonyloxytryptamine oxalate, is advancing rapidly, showing potential in promoting repair of spinal cord and peripheral nerve injuries in animal models.

Furthermore, the human inhibitory SIGLEC receptor SIGLEC-11 has been shown to alleviate immune-mediated neuronal cell damage. SIGLEC-11, when ectopically expressed in cultured mouse microglia, prevents microglial phagocytosis of apoptotic neuronal material and reduces lipopolysaccharide (LPS)-induced inflammation [248]. The SIGLEC-11 ligand, polySia with an average degree of polymerization of 20 (polySia avDP20), also suppresses phagocyte and macrophage oxidative burst functions in LPS-exposed environments [249], highlighting the potential of polySia-based and SIGLEC agonistic approaches in limiting acute nervous system injury.

### Effects of sialic acid on stabilizing nanocarriers

To stabilize nanocarriers in the bloodstream, polysialylation has been employed using compounds bearing one or more sialic acid moieties. Most research has focused on incorporating sialic acid onto the surfaces of liposomes. Ganglioside GM1 is an early application of a surface modifier used to stabilize nanocarriers [250]. It can be integrated into the liposome bilayer, presenting sialic acid molecules on the nanocarrier surface. The use of GM1 can significantly reduce reticuloendothelial system uptake of liposomes in mice [251]. The effectiveness of this approach is influenced by liposome size [252], with liposomes between 90 and 200 nm being most suitable for tumor cell accumulation [253]. Other chemical moieties, such as fucose residues, can also affect sialic acid activity, with the presence of fucose increasing reticuloendothelial system uptake of glycoprotein-conjugated liposomes. In addition, pH levels can influence the stability and drug release profile of sialic acid-modified liposomal formulations [254].

## Conclusions and perspectives

Sialic acids participate in various facets of cell biology. On the cell surface or glycocalyx, sialic acids act as cytoprotectors, with fundamental functions that include signal transduction via receptors like SIGLECs and selectins, and anti-molecular adhesion due to their negatively charged surface. Sialylation plays a crucial role in immune system regulation, including complement activation, leukocyte trafficking, dendritic cell maturation, and modulation of antibody effects. It also contributes to maintaining stem cell properties and fertility. However, sialic acids can be exploited by pathogens and malignant cells. Dysregulation of sialylation has been linked to cancer progression, CNS diseases, viral infections, atherosclerosis, and CVDs. Thus, novel therapeutic strategies targeting the sialylation process are being developed. Inhibitors of sialyltransferases, selectins, and SIGLECs have been tested in animal models of cancer, and sialidase-conjugated antibodies for anti-cancer therapy are entering clinical trials. Modifying drug delivery systems with sialic acid holds potential for treating stroke-induced brain reperfusion injury. In addition, strategies that mimic sialidase or block interactions between sialic acid and viral surface glycoproteins are under investigation for antiviral therapy. Blocking sialic acid-binding receptors and supplementing polysialic acid may offer promising methods for CNS disorder treatment.

Despite advancements in sialylation research, numerous questions remain. First, while sialic acids are ubiquitous, studies on their pathophysiological roles are limited to a few systems. Most research focuses on cancer biology and the nervous system, with potential roles in other systems (e.g., digestive and urinary systems) yet to be explored. Second, although there is a link between aberrant sialylation and psychiatric diseases, the specific mechanisms are not well understood. Third, in the fields such as CVDs, prior researches are primarily focused on understanding sialylation’s role in pathological or physiological processes, with its therapeutic potential remaining unclear. Lastly, most current approaches targeting sialylation are in the pre-clinical stage.

## Supplementary information

Agents Targeting Sialylation for Disease Treatment

## References

[1] Sprenger N, Duncan PI. Sialic acid utilization. Adv Nutr. 2012;3:392S–7S. 22585917 10.3945/an.111.001479 PMC3649475

[2] Corfield AP, Wember M, Schauer R, Rott R. The specificity of viral sialidases. The use of oligosaccharide substrates to probe enzymic characteristics and strain-specific differences. Eur J Biochem. 1982;124:521–5. 7106104

[3] Blix G. Über die Kohlenhydratgruppen des Submaxillarismucins. Biol Chem. 1936;240:43–54.

[4] Angata T, Varki A. Chemical diversity in the sialic acids and related alpha-keto acids: an evolutionary perspective. Chem Rev. 2002;102:439–69. 11841250 10.1021/cr000407m

[5] Varki A. N-glycolylneuraminic acid deficiency in humans. Biochimie. 2001;83:615–22. 11522390 10.1016/s0300-9084(01)01309-8

[6] Tangvoranuntakul P, Gagneux P, Diaz S, Bardor M, Varki N, Varki A, et al. Human uptake and incorporation of an immunogenic nonhuman dietary sialic acid. Proc Natl Acad Sci USA. 2003;100:12045–50. 14523234 10.1073/pnas.2131556100 PMC218710

[7] Padler-Karavani V, Yu H, Cao H, Chokhawala H, Karp F, Varki N, et al. Diversity in specificity, abundance, and composition of anti-Neu5Gc antibodies in normal humans: potential implications for disease. Glycobiology. 2008;18:818–30. 18669916 10.1093/glycob/cwn072 PMC2586336

[8] Li F, Ding J. Sialylation is involved in cell fate decision during development, reprogramming and cancer progression. Protein Cell. 2019;10:550–65. 30478534 10.1007/s13238-018-0597-5 PMC6626595

[9] Chen X, Varki A. Advances in the biology and chemistry of sialic acids. ACS Chem Biol. 2010;5:163–76. 20020717 10.1021/cb900266r PMC2825284

[10] Varki A. Glycan-based interactions involving vertebrate sialic-acid-recognizing proteins. Nature. 2007;446:1023–9. 17460663 10.1038/nature05816

[11] Cohen M, Varki A. The sialome–far more than the sum of its parts. Omics. J Integr Biol. 2010;14:455–64. 10.1089/omi.2009.0148 20726801

[12] Pshezhetsky AV, Hinek A. Where catabolism meets signalling: neuraminidase 1 as a modulator of cell receptors. Glycoconj J. 2011;28:441–52. 21928149 10.1007/s10719-011-9350-5

[13] Ain KB, Mori Y, Refetoff S. Reduced clearance rate of thyroxine-binding globulin (TBG) with increased sialylation: a mechanism for estrogen-induced elevation of serum TBG concentration. J Clin Endocrinol Metab. 1987;65:689–96. 3116030 10.1210/jcem-65-4-689

[14] Elliott S, Egrie J, Browne J, Lorenzini T, Busse L, Rogers N, et al. Control of rHuEPO biological activity: the role of carbohydrate. Exp Hematol. 2004;32:1146–55. 15588939 10.1016/j.exphem.2004.08.004

[15] Keyt BA, Paoni NF, Refino CJ, Berleau L, Nguyen H, Chow A, et al. A faster-acting and more potent form of tissue plasminogen activator. Proc Natl Acad Sci USA. 1994;91:3670–4. 8170967 10.1073/pnas.91.9.3670 PMC43643

[16] Kimura R, Miller WM. Glycosylation of CHO-derived recombinant tPA produced under elevated pCO2. Biotechnol Prog. 1997;13:311–7. 9190082 10.1021/bp9700162

[17] Raju TS, Lang SE. Diversity in structure and functions of antibody sialylation in the Fc. Curr Opin Biotechnol. 2014;30:147–52. 25032906 10.1016/j.copbio.2014.06.014

[18] Varki A. Sialic acids in human health and disease. Trends Mol Med. 2008;14:351–60. 18606570 10.1016/j.molmed.2008.06.002 PMC2553044

[19] Born GV, Palinski W. Unusually high concentrations of sialic acids on the surface of vascular endothelia. Br J Exp Pathol. 1985;66:543–9. 4063159 PMC2042046

[20] Weber KS, Alon R, Klickstein LB. Sialylation of ICAM-2 on platelets impairs adhesion of leukocytes via LFA-1 and DC-SIGN. Inflammation. 2004;28:177–88. 15673159 10.1023/b:ifla.0000049042.73926.eb

[21] Zhuravleva MA, Trandem K, Sun PD. Structural implications of Siglec-5-mediated sialoglycan recognition. J Mol Biol. 2008;375:437–47. 18022638 10.1016/j.jmb.2007.10.009 PMC2245879

[22] Hiruma Y, Hirai T, Tsuda E. Siglec-15, a member of the sialic acid-binding lectin, is a novel regulator for osteoclast differentiation. Biochem Biophys Res Commun. 2011;409:424–9. 21586272 10.1016/j.bbrc.2011.05.015

[23] Lühn K, Wild MK. Human deficiencies of fucosylation and sialylation affecting selectin ligands. Semin Immunopathol. 2012;34:383–99. 22461019 10.1007/s00281-012-0304-1

[24] Miyagi T, Yamaguchi K. Mammalian sialidases: physiological and pathological roles in cellular functions. Glycobiology. 2012;22:880–96. 22377912 10.1093/glycob/cws057

[25] Glanz VY, Myasoedova VA, Grechko AV, Orekhov AN. Sialidase activity in human pathologies. Eur J Pharmacol. 2019;842:345–50. 30439363 10.1016/j.ejphar.2018.11.014

[26] Bochner BS, Zimmermann N. Role of siglecs and related glycan-binding proteins in immune responses and immunoregulation. J Allergy Clin Immunol. 2015;135:598–608. 25592986 10.1016/j.jaci.2014.11.031 PMC4355302

[27] Zhou X, Yang G, Guan F. Biological functions and analytical strategies of sialic acids in tumor. Cells. 2020;9:273. 31979120 10.3390/cells9020273 PMC7072699

[28] Yang K, Yang Z, Chen X, Li W. The significance of sialylation on the pathogenesis of Alzheimer’s disease. Brain Res Bull. 2021;173:116–23. 33991608 10.1016/j.brainresbull.2021.05.009

[29] Stadlmann J, Pabst M, Altmann F. Analytical and functional aspects of antibody sialylation. J Clin Immunol. 2010;30:15–9. 10.1007/s10875-010-9409-2 PMC2883086 20390325

[30] Blaum BS, Hannan JP, Herbert AP, Kavanagh D, Uhrín D, Stehle T. Structural basis for sialic acid-mediated self-recognition by complement factor H. Nat Chem Biol. 2015;11:77–82. 25402769 10.1038/nchembio.1696

[31] Varki A, Gagneux P. Multifarious roles of sialic acids in immunity. Ann N. Y Acad Sci. 2012;1253:16–36. 22524423 10.1111/j.1749-6632.2012.06517.x PMC3357316

[32] Ram S, Sharma AK, Simpson SD, Gulati S, McQuillen DP, Pangburn MK, et al. A novel sialic acid binding site on factor H mediates serum resistance of sialylated Neisseria gonorrhoeae. J Exp Med. 1998;187:743–52. 9480984 10.1084/jem.187.5.743 PMC2212180

[33] Johnston JW, Coussens NP, Allen S, Houtman JC, Turner KH, Zaleski A, et al. Characterization of the N-acetyl-5-neuraminic acid-binding site of the extracytoplasmic solute receptor (SiaP) of nontypeable Haemophilus influenzae strain 2019. J Biol Chem. 2008;283:855–65. 17947229 10.1074/jbc.M706603200

[34] Ram S, Lewis LA, Agarwal S. Meningococcal group W-135 and Y capsular polysaccharides paradoxically enhance activation of the alternative pathway of complement. J Biol Chem. 2011;286:8297–307. 21245150 10.1074/jbc.M110.184838 PMC3048715

[35] Nydegger UE, Fearon DT, Austen KF. Autosomal locus regulates inverse relationship between sialic acid content and capacity of mouse erythrocytes to activate human alternative complement pathway. Proc Natl Acad Sci USA. 1978;75:6078–82. 282625 10.1073/pnas.75.12.6078 PMC393121

[36] Shi WX, Chammas R, Varki NM, Powell L, Varki A. Sialic acid 9-O-acetylation on murine erythroleukemia cells affects complement activation, binding to I-type lectins, and tissue homing. J Biol Chem. 1996;271:31526–32. 8940168 10.1074/jbc.271.49.31526

[37] Kjaer TR, Hansen AG, Sørensen UB, Nielsen O, Thiel S, Jensenius JC. Investigations on the pattern recognition molecule M-ficolin: quantitative aspects of bacterial binding and leukocyte association. J Leukoc Biol. 2011;90:425–37. 21730084 10.1189/jlb.0411201

[38] Honoré C, Rørvig S, Hummelshøj T, Skjoedt MO, Borregaard N, Garred P. Tethering of Ficolin-1 to cell surfaces through recognition of sialic acid by the fibrinogen-like domain. J Leukoc Biol. 2010;88:145–58. 20400674 10.1189/jlb.1209802

[39] Gout E, Garlatti V, Smith DF, Lacroix M, Dumestre-Pérard C, Lunardi T, et al. Carbohydrate recognition properties of human ficolins: glycan array screening reveals the sialic acid binding specificity of M-ficolin. J Biol Chem. 2010;285:6612–22. 20032467 10.1074/jbc.M109.065854 PMC2825457

[40] Crocker PR, Paulson JC, Varki A. Siglecs and their roles in the immune system. Nat Rev Immunol. 2007;7:255–66. 17380156 10.1038/nri2056

[41] Pillai S, Netravali IA, Cariappa A, Mattoo H. Siglecs and immune regulation. Annu Rev Immunol. 2012;30:357–92. 22224769 10.1146/annurev-immunol-020711-075018 PMC3781015

[42] Chen GY, Chen X, King S, Cavassani KA, Cheng J, Zheng X, et al. Amelioration of sepsis by inhibiting sialidase-mediated disruption of the CD24-SiglecG interaction. Nat Biotechnol. 2011;29:428–35. 21478876 10.1038/nbt.1846 PMC4090080

[43] Spence S, Greene MK, Fay F, Hams E, Saunders SP, Hamid U, et al. Targeting Siglecs with a sialic acid-decorated nanoparticle abrogates inflammation. Sci Transl Med. 2015;7:303 ra140. 10.1126/scitranslmed.aab3459 26333936

[44] Lünemann JD, von Gunten S, Neumann H. Targeting sialylation to treat central nervous system diseases. Trends Pharmacol Sci. 2021;42:998–1008. 34607695 10.1016/j.tips.2021.09.002

[45] Toubai T, Hou G, Mathewson N, Liu C, Wang Y, Oravecz-Wilson K, et al. Siglec-G-CD24 axis controls the severity of graft-versus-host disease in mice. Blood. 2014;123:3512–23. 24695850 10.1182/blood-2013-12-545335 PMC4041170

[46] Lübbers J, Rodríguez E, van Kooyk Y. Modulation of immune tolerance via siglec-sialic acid interactions. Front Immunol. 2018;9:2807. 30581432 10.3389/fimmu.2018.02807 PMC6293876

[47] Angata T, Margulies EH, Green ED, Varki A. Large-scale sequencing of the CD33-related Siglec gene cluster in five mammalian species reveals rapid evolution by multiple mechanisms. Proc Natl Acad Sci USA. 2004;101:13251–6. 15331780 10.1073/pnas.0404833101 PMC516556

[48] Makarava N, Baskakov IV. Role of sialylation of N-linked glycans in prion pathogenesis. Cell Tissue Res. 2022;392:201–14. 35088180 10.1007/s00441-022-03584-2 PMC9329487

[49] Zhang Y, Ohkuri T, Wakita D, Narita Y, Chamoto K, Kitamura H, et al. Sialyl lewisx antigen-expressing human CD4+ T and CD8+ T cells as initial immune responders in memory phenotype subsets. J Leukoc Biol. 2008;84:730–5. 18523229 10.1189/jlb.0907599

[50] Sperandio M. The expanding role of α2‐3 sialylation for leukocyte trafficking in vivo. Ann N. Y Acad Sci. 2012;1253:201–5. 22257379 10.1111/j.1749-6632.2011.06271.x

[51] Sperandio M, Gleissner CA, Ley K. Glycosylation in immune cell trafficking. Immunol Rev. 2009;230:97–113. 19594631 10.1111/j.1600-065X.2009.00795.x PMC2745114

[52] Scallon BJ, Tam SH, McCarthy SG, Cai AN, Raju TS. Higher levels of sialylated Fc glycans in immunoglobulin G molecules can adversely impact functionality. Mol Immunol. 2007;44:1524–34. 17045339 10.1016/j.molimm.2006.09.005

[53] Raju TS, Briggs JB, Borge SM, Jones AJ. Species-specific variation in glycosylation of IgG: evidence for the species-specific sialylation and branch-specific galactosylation and importance for engineering recombinant glycoprotein therapeutics. Glycobiology. 2000;10:477–86. 10764836 10.1093/glycob/10.5.477

[54] Orczyk-Pawiłowicz M, Augustyniak D, Hirnle L, Kątnik-Prastowska I. Degree of sialylation and fucosylation of plasma and amniotic immunoglobulin G changes progressively during normal pregnancy. Prenat Diagn. 2012;32:432–9. 22495687 10.1002/pd.3832

[55] Anthony RM, Nimmerjahn F, Ashline DJ, Reinhold VN, Paulson JC, Ravetch JV. Recapitulation of IVIG anti-inflammatory activity with a recombinant IgG Fc. Science. 2008;320:373–6. 18420934 10.1126/science.1154315 PMC2409116

[56] Anthony RM, Ravetch JV. A novel role for the IgG Fc glycan: the anti-inflammatory activity of sialylated IgG Fcs. J Clin Immunol. 2010;30:S9–14. 20480216 10.1007/s10875-010-9405-6

[57] Schwab I, Nimmerjahn F. Intravenous immunoglobulin therapy: how does IgG modulate the immune system? Nat Rev Immunol. 2013;13:176–89. 23411799 10.1038/nri3401

[58] Baerenwaldt A, Biburger M, Nimmerjahn F. Mechanisms of action of intravenous immunoglobulins. Expert Rev Clin Immunol. 2010;6:425–34. 20441428 10.1586/eci.10.9

[59] Wu RQ, Lao XM, Chen DP, Qin H, Mu M, Cao WJ, et al. Immune checkpoint therapy-elicited sialylation of IgG antibodies impairs antitumorigenic type I interferon responses in hepatocellular carcinoma. Immunity. 2023;56:180–92.e11. 36563676 10.1016/j.immuni.2022.11.014

[60] Balneger N, Cornelissen LAM, Wassink M, Moons SJ, Boltje TJ, Bar-Ephraim YE, et al. Sialic acid blockade in dendritic cells enhances CD8(+) T cell responses by facilitating high-avidity interactions. Cell Mol Life Sci. 2022;79:98. 35089436 10.1007/s00018-021-04027-x PMC8799591

[61] Crespo HJ, Cabral MG, Teixeira AV, Lau JT, Trindade H, Videira PA. Effect of sialic acid loss on dendritic cell maturation. Immunology. 2009;128:e621–31. 19740323 10.1111/j.1365-2567.2009.03047.x PMC2753891

[62] Silva M, Silva Z, Marques G, Ferro T, Gonçalves M, Monteiro M, et al. Sialic acid removal from dendritic cells improves antigen cross-presentation and boosts anti-tumor immune responses. Oncotarget. 2016;7:41053–66. 27203391 10.18632/oncotarget.9419 PMC5173042

[63] Cabral MG, Silva Z, Ligeiro D, Seixas E, Crespo H, Carrascal MA, et al. The phagocytic capacity and immunological potency of human dendritic cells is improved by α2,6-sialic acid deficiency. Immunology. 2013;138:235–45. 23113614 10.1111/imm.12025 PMC3573277

[64] Villanueva-Cabello TM, Gutiérrez-Valenzuela LD, Salinas-Marín R, López-Guerrero DV, Martínez-Duncker I. Polysialic Acid in the Immune System. Front Immunol. 2021;12:823637. 35222358 10.3389/fimmu.2021.823637 PMC8873093

[65] Nabatov AA, Raginov IS. The DC-SIGN-CD56 interaction inhibits the anti-dendritic cell cytotoxicity of CD56 expressing cells. Infect Agents Cancer. 2015;10:49. 10.1186/s13027-015-0043-8 PMC4676137 26692894

[66] Wang YC, Peterson SE, Loring JF. Protein post-translational modifications and regulation of pluripotency in human stem cells. Cell Res. 2014;24:143–60. 24217768 10.1038/cr.2013.151 PMC3915910

[67] Melo-Braga MN, Schulz M, Liu Q, Swistowski A, Palmisano G, Engholm-Keller K, et al. Comprehensive quantitative comparison of the membrane proteome, phosphoproteome, and sialiome of human embryonic and neural stem cells. Mol Cell Proteom. 2014;13:311–28. 10.1074/mcp.M112.026898 PMC3879623 24173317

[68] Yi S, Feng Y, Wang Y, Ma F. Sialylation: fate decision of mammalian sperm development, fertilization, and male fertility. Biol Reprod. 2023;109:137–55. 37379321 10.1093/biolre/ioad067

[69] Ghaderi D, Springer SA, Ma F, Cohen M, Secrest P, Taylor RE, et al. Sexual selection by female immunity against paternal antigens can fix loss of function alleles. Proc Natl Acad Sci USA. 2011;108:17743–8. 21987817 10.1073/pnas.1102302108 PMC3203784

[70] Janiszewska E, Kokot I, Kmieciak A, Stelmasiak Z, Gilowska I, Faundez R, et al. The association between clusterin sialylation degree and levels of oxidative–antioxidant balance markers in seminal plasmas and blood sera of male partners with abnormal sperm parameters. Int J Mol Sci. 2022;23:10598. 36142505 10.3390/ijms231810598 PMC9501354

[71] Bernal A, Torres J, Reyes A, Rosado A. Presence and regional distribution of sialyl transferase in the epididymis of the rat. Biol Reprod. 1980;23:290–3. 7417673 10.1095/biolreprod23.2.290

[72] Feng Y, Wang L, Wu YL, Liu HH, Ma F. Roles of sialic acids in sperm maturation and capacitation and sperm-egg recognition. Zhonghua Nan Ke Xue. 2016;22:944–8. 29278479

[73] Alkhodair K, Almhanna H, McGetrick J, Gedair S, Gallagher ME, Fernandez-Fuertes B, et al. Siglec expression on the surface of human, bull and ram sperm. Reproduction. 2018;155:361–71. 29581386 10.1530/REP-17-0475

[74] Kambara Y, Shiba K, Yoshida M, Sato C, Kitajima K, Shingyoji C. Mechanism regulating Ca2+-dependent mechanosensory behaviour in sea urchin spermatozoa. Cell Struct Funct. 2011;36:69–82. 21358125 10.1247/csf.10020

[75] Acott TS, Hoskins DD. Bovine sperm forward motility protein. Partial purification and characterization. J Biol Chem. 1978;253:6744–50. 211130

[76] Zhai YJ, Feng Y, Ma X, Ma F. Defensins: defenders of human reproductive health. Hum Reprod update. 2023;29:126–54. 36130055 10.1093/humupd/dmac032 PMC9825273

[77] Tollner TL, Bevins CL, Cherr GN. Multifunctional glycoprotein DEFB126–a curious story of defensin-clad spermatozoa. Nat Rev Urol. 2012;9:365–75. 22710670 10.1038/nrurol.2012.109

[78] Hänsch M, Simon P, Schön J, Kaese M, Braun BC, Jewgenow K, et al. Polysialylation of NCAM correlates with onset and termination of seasonal spermatogenesis in roe deer. Glycobiology. 2014;24:488–93. 24663385 10.1093/glycob/cwu023

[79] Kanato Y, Kitajima K, Sato C. Direct binding of polysialic acid to a brain-derived neurotrophic factor depends on the degree of polymerization. Glycobiology. 2008;18:1044–53. 18796648 10.1093/glycob/cwn084

[80] Li C, Zhou X. The potential roles of neurotrophins in male reproduction. Reproduction. 2013;145:R89–95. 23404847 10.1530/REP-12-0466

[81] Simon P, Feuerstacke C, Kaese M, Saboor F, Middendorff R, Galuska SP. Polysialylation of NCAM characterizes the proliferation period of contractile elements during postnatal development of the epididymis. PloS ONE. 2015;10:e0123960. 25822229 10.1371/journal.pone.0123960 PMC4379024

[82] Simon P, Bäumner S, Busch O, Röhrich R, Kaese M, Richterich P, et al. Polysialic acid is present in mammalian semen as a post-translational modification of the neural cell adhesion molecule NCAM and the polysialyltransferase ST8SiaII. J Biol Chem. 2013;288:18825–33. 23671285 10.1074/jbc.M113.451112 PMC3696658

[83] Aakhus AM, Stavem P, Hovig T, Pedersen TM, Solum NO. Studies on a patient with thrombocytopenia, giant platelets and a platelet membrane glycoprotein Ib with reduced amount of sialic acid. Br J Haematol. 1990;74:320–9. 2334639 10.1111/j.1365-2141.1990.tb02590.x

[84] Gröttum KA, Solum NO. Congenital thrombocytopenia with giant platelets: a defect in the platelet membrane. Br J Haematol. 1969;16:277–90. 4893927 10.1111/j.1365-2141.1969.tb00402.x

[85] Kunishima S, Kamiya T, Saito H. Genetic abnormalities of Bernard-Soulier syndrome. Int J Hematol. 2002;76:319–27. 12463594 10.1007/BF02982690

[86] Ward S, O’Sullivan JM, O’Donnell JS. von Willebrand factor sialylation-A critical regulator of biological function. J Thromb Haemost. 2019;17:1018–29. 31055873 10.1111/jth.14471

[87] Federici AB, De Romeuf C, De Groot PG, Samor B, Lombardi R, D’Alessio P, et al. Adhesive properties of the carbohydrate-modified von Willebrand factor (CHO-vWF). Blood. 1988;71:947–52. 3128350

[88] Berkowitz SD, Federici AB. Sialic acid prevents loss of large von Willebrand factor multimers by protecting against amino-terminal proteolytic cleavage. Blood. 1988;72:1790–6. 2460162

[89] Vajaria BN, Patel KR, Begum R, Patel PS. Sialylation: an avenue to target cancer cells. Pathol Oncol Res. 2015;22:443–7. 26685886 10.1007/s12253-015-0033-6

[90] Reily C, Stewart TJ, Renfrow MB, Novak J. Glycosylation in health and disease. Nat Rev Nephrol. 2019;15:346–66. 30858582 10.1038/s41581-019-0129-4 PMC6590709

[91] Dobie C, Skropeta D. Insights into the role of sialylation in cancer progression and metastasis. Br J Cancer. 2020;124:76–90. 33144696 10.1038/s41416-020-01126-7 PMC7782833

[92] Shah MH, Telang SD, Shah PM, Patel PS. Tissue and serum alpha 2-3- and alpha 2-6-linkage specific sialylation changes in oral carcinogenesis. Glycoconj J. 2008;25:279–90. 18158621 10.1007/s10719-007-9086-4

[93] Cylwik B, Chrostek L, Szmitkowski M. Diagnostic value of total and lipid-bound sialic acid in malignancies. Pol Merkur Lekarski. 2005;19:237–41. 16245443

[94] Bose KS, Gokhale PV, Dwivedi S, Singh M. Quantitative evaluation and correlation of serum glycoconjugates: protein bound hexoses, sialic acid and fucose in leukoplakia, oral sub mucous fibrosis and oral cancer. J Nat Sci Biol Med. 2013;4:122–5. 23633847 10.4103/0976-9668.107275 PMC3633261

[95] Zhang Z, Wuhrer M, Holst S. Serum sialylation changes in cancer. Glycoconj J. 2018;35:139–60. 29680984 10.1007/s10719-018-9820-0 PMC5916985

[96] Huang J, Huang J, Zhang G. Insights into the role of sialylation in cancer metastasis, immunity, and therapeutic opportunity. Cancers. 2022;14:5840. 36497322 10.3390/cancers14235840 PMC9737300

[97] Britain CM, Bhalerao N, Silva AD, Chakraborty A, Buchsbaum DJ, Crowley MR, et al. Glycosyltransferase ST6Gal-I promotes the epithelial to mesenchymal transition in pancreatic cancer cells. J Biol Chem. 2021;296:100034. 33148698 10.1074/jbc.RA120.014126 PMC7949065

[98] Rao TC, Ma VP, Blanchard A, Urner TM, Grandhi S, Salaita K, et al. EGFR activation attenuates the mechanical threshold for integrin tension and focal adhesion formation. J cell Sci. 2020;133:jcs238840. 32546532 10.1242/jcs.238840 PMC7358133

[99] Rao TC, Beggs RR, Ankenbauer KE, Hwang J, Ma VP, Salaita K, et al. ST6Gal-I-mediated sialylation of the epidermal growth factor receptor modulates cell mechanics and enhances invasion. J Biol Chem. 2022;298:101726. 35157848 10.1016/j.jbc.2022.101726 PMC8956946

[100] Christie DR, Shaikh FM. Lucas JAt, Lucas JA, 3rd, Bellis SL. ST6Gal-I expression in ovarian cancer cells promotes an invasive phenotype by altering integrin glycosylation and function. J Ovarian Res. 2008;1:3. 19014651 10.1186/1757-2215-1-3 PMC2584051

[101] Ou L, He X, Liu N, Song Y, Li J, Gao L, et al. Sialylation of FGFR1 by ST6Gal‑I overexpression contributes to ovarian cancer cell migration and chemoresistance. Mol Med Rep. 2020;21:1449–60. 32016470 10.3892/mmr.2020.10951 PMC7003046

[102] Zhang X, Dou P, Akhtar ML, Liu F, Hu X, Yang L, et al. NEU4 inhibits motility of HCC cells by cleaving sialic acids on CD44. Oncogene. 2021;40:5427–40. 34282273 10.1038/s41388-021-01955-7

[103] Cazet A, Julien S, Bobowski M, Krzewinski-Recchi MA, Harduin-Lepers A, Groux-Degroote S, et al. Consequences of the expression of sialylated antigens in breast cancer. Carbohydr Res. 2010;345:1377–83. 20231016 10.1016/j.carres.2010.01.024

[104] Ozaki H, Matsuzaki H, Ando H, Kaji H, Nakanishi H, Ikehara Y, et al. Enhancement of metastatic ability by ectopic expression of ST6GalNAcI on a gastric cancer cell line in a mouse model. Clin Exp Metastasis. 2012;29:229–38. 22228572 10.1007/s10585-011-9445-1 PMC3275730

[105] Elkashef SM, Allison SJ, Sadiq M, Basheer HA, Ribeiro Morais G, Loadman PM, et al. Polysialic acid sustains cancer cell survival and migratory capacity in a hypoxic environment. Sci Rep. 2016;6:33026. 27611649 10.1038/srep33026 PMC5017143

[106] Rosa P, Scibetta S, Pepe G, Mangino G, Capocci L, Moons SJ, et al. Polysialic acid sustains the hypoxia-induced migration and undifferentiated state of human glioblastoma cells. Int J Mol Sci. 2022;23:9563. 36076963 10.3390/ijms23179563 PMC9455737

[107] Ferrer CM, Reginato MJ. Sticking to sugars at the metastatic site: sialyltransferase ST6GalNAc2 acts as a breast cancer metastasis suppressor. Cancer Discov. 2014;4:275–7. 24596201 10.1158/2159-8290.CD-14-0075 PMC3964769

[108] Gu Y, Zhang J, Mi W, Yang J, Han F, Lu X, et al. Silencing of GM3 synthase suppresses lung metastasis of murine breast cancer cells. Breast cancer Res. 2008;10:R1. BCR 18171481 10.1186/bcr1841 PMC2374951

[109] Napoletano C, Steentoff C, Battisti F, Ye Z, Rahimi H, Zizzari IG, et al. Investigating patterns of immune interaction in ovarian cancer: probing the O-glycoproteome by the macrophage galactose-like C-type lectin (MGL). Cancers. 2020;12:2841. 33019700 10.3390/cancers12102841 PMC7600217

[110] Läubli H, Varki A. Sialic acid-binding immunoglobulin-like lectins (Siglecs) detect self-associated molecular patterns to regulate immune responses. Cell Mol Life Sci. 2020;77:593–605. 31485715 10.1007/s00018-019-03288-x PMC7942692

[111] Kim C-H. CD33 and CD33-related siglecs in pathogen recognition and endocytosis of DC in the innate immune system. In: Kim C-H, editor. Glycobiology of innate immunology. Singapore: Springer Singapore; 2022. p. 631–56.

[112] Hiltbold EM, Vlad AM, Ciborowski P, Watkins SC, Finn OJ. The mechanism of unresponsiveness to circulating tumor antigen MUC1 is a block in intracellular sorting and processing by dendritic cells. J Immunol. 2000;165:3730–41. 11034378 10.4049/jimmunol.165.7.3730

[113] Brunngraber EG, Witting LA, Haberland C, Brown B. Glycoproteins in Tay-sachs disease: isolation and carbohydrate composition of glycopeptides. Brain Res. 1972;38:151–62. 4259417 10.1016/0006-8993(72)90596-3

[114] Angata K, Fukuda M. Roles of polysialic acid in migration and differentiation of neural stem cells. Methods Enzymol. 2010;479:25–36. 20816158 10.1016/S0076-6879(10)79002-9

[115] Puigdellívol M, Allendorf DH, Brown GC. Sialylation and galectin-3 in microglia-mediated neuroinflammation and neurodegeneration. Front Cell Neurosci. 2020;14:162. 32581723 10.3389/fncel.2020.00162 PMC7296093

[116] Abe C, Yi Y, Hane M, Kitajima K, Sato C. Acute stress-induced change in polysialic acid levels mediated by sialidase in mouse brain. Sci Rep. 2019;9:9950. 31289315 10.1038/s41598-019-46240-6 PMC6616613

[117] Minami A, Saito M, Mamada S, Ieno D, Hikita T, Takahashi T, et al. Role of sialidase in long-term potentiation at mossy fiber-CA3 synapses and hippocampus-dependent spatial memory. PloS one. 2016;11:e0165257. 27783694 10.1371/journal.pone.0165257 PMC5081204

[118] Chen Y, Zhao S, Fan Z, Li Z, Zhu Y, Shen T, et al. Metformin attenuates plaque-associated tau pathology and reduces amyloid-β burden in APP/PS1 mice. Alzheimer’s Res Ther. 2021;13:40. 33563332 10.1186/s13195-020-00761-9 PMC7871393

[119] Nakagawa K, Kitazume S, Oka R, Maruyama K, Saido TC, Sato Y, et al. Sialylation enhances the secretion of neurotoxic amyloid-beta peptides. J Neurochem. 2006;96:924–33. 16412100 10.1111/j.1471-4159.2005.03595.x

[120] Lewcock JW, Schlepckow K, Di Paolo G, Tahirovic S, Monroe KM, Haass C. Emerging microglia biology defines novel therapeutic approaches for Alzheimer’s disease. Neuron. 2020;108:801–21. 33096024 10.1016/j.neuron.2020.09.029

[121] Comi G, Bar-Or A, Lassmann H, Uccelli A, Hartung HP, Montalban X, et al. Role of B cells in multiple sclerosis and related disorders. Ann Neurol. 2021;89:13–23. 33091175 10.1002/ana.25927 PMC8007167

[122] Duong BH, Tian H, Ota T, Completo G, Han S, Vela JL, et al. Decoration of T-independent antigen with ligands for CD22 and Siglec-G can suppress immunity and induce B cell tolerance in vivo. J Exp Med. 2010;207:173–87. 20038598 10.1084/jem.20091873 PMC2812539

[123] Macauley MS, Pfrengle F, Rademacher C, Nycholat CM, Gale AJ, von Drygalski A, et al. Antigenic liposomes displaying CD22 ligands induce antigen-specific B cell apoptosis. J Clin Investig. 2013;123:3074–83. 23722906 10.1172/JCI69187 PMC3706185

[124] McAuley EZ, Scimone A, Tiwari Y, Agahi G, Mowry BJ, Holliday EG, et al. Identification of sialyltransferase 8B as a generalized susceptibility gene for psychotic and mood disorders on chromosome 15q25-26. PloS one. 2012;7:e38172. 22693595 10.1371/journal.pone.0038172 PMC3364966

[125] El Maarouf A, Petridis AK, Rutishauser U. Use of polysialic acid in repair of the central nervous system. Proc Natl Acad Sci USA. 2006;103:16989–94. 17075041 10.1073/pnas.0608036103 PMC1636566

[126] Zhang Y, Zhang X, Yeh J, Richardson P, Bo X. Engineered expression of polysialic acid enhances Purkinje cell axonal regeneration in L1/GAP-43 double transgenic mice. Eur J Neurosci. 2007;25:351–61. 17284175 10.1111/j.1460-9568.2007.05311.x

[127] Zhang Y, Zhang X, Wu D, Verhaagen J, Richardson PM, Yeh J, et al. Lentiviral-mediated expression of polysialic acid in spinal cord and conditioning lesion promote regeneration of sensory axons into spinal cord. Mol Ther. 2007;15:1796–804. 17551503 10.1038/sj.mt.6300220

[128] Papastefanaki F, Chen J, Lavdas AA, Thomaidou D, Schachner M, Matsas R. Grafts of Schwann cells engineered to express PSA-NCAM promote functional recovery after spinal cord injury. Brain. 2007;130:2159–74. 17626035 10.1093/brain/awm155

[129] Charles P, Hernandez MP, Stankoff B, Aigrot MS, Colin C, Rougon G, et al. Negative regulation of central nervous system myelination by polysialylated-neural cell adhesion molecule. Proc Natl Acad Sci USA. 2000;97:7585–90. 10840047 10.1073/pnas.100076197 PMC16589

[130] Charles P, Reynolds R, Seilhean D, Rougon G, Aigrot MS, Niezgoda A, et al. Re-expression of PSA-NCAM by demyelinated axons: an inhibitor of remyelination in multiple sclerosis? Brain. 2002;125:1972–9. 12183343 10.1093/brain/awf216

[131] Franceschini I, Vitry S, Padilla F, Casanova P, Tham TN, Fukuda M, et al. Migrating and myelinating potential of neural precursors engineered to overexpress PSA-NCAM. Mol Cell Neurosci. 2004;27:151–62. 15485771 10.1016/j.mcn.2004.05.006

[132] Mehanna A, Jakovcevski I, Acar A, Xiao M, Loers G, Rougon G, et al. Polysialic acid glycomimetic promotes functional recovery and plasticity after spinal cord injury in mice. Mol Ther. 2010;18:34–43. 19826404 10.1038/mt.2009.235 PMC2839208

[133] Saini V, Lutz D, Kataria H, Kaur G, Schachner M, Loers G. The polysialic acid mimetics 5-nonyloxytryptamine and vinorelbine facilitate nervous system repair. Sci Rep. 2016;6:26927. 27324620 10.1038/srep26927 PMC4914991

[134] Bushman J, Mishra B, Ezra M, Gul S, Schulze C, Chaudhury S, et al. Tegaserod mimics the neurostimulatory glycan polysialic acid and promotes nervous system repair. Neuropharmacology. 2014;79:456–66. 24067923 10.1016/j.neuropharm.2013.09.014 PMC4618794

[135] Jungnickel J, Eckhardt M, Haastert-Talini K, Claus P, Bronzlik P, Lipokatic-Takacs E, et al. Polysialyltransferase overexpression in Schwann cells mediates different effects during peripheral nerve regeneration. Glycobiology. 2012;22:107–15. 21840969 10.1093/glycob/cwr113

[136] Mehanna A, Mishra B, Kurschat N, Schulze C, Bian S, Loers G, et al. Polysialic acid glycomimetics promote myelination and functional recovery after peripheral nerve injury in mice. Brain. 2009;132:1449–62. 19454531 10.1093/brain/awp128

[137] Kalotra S, Saini V, Singh H, Sharma A, Kaur G. 5-Nonyloxytryptamine oxalate-embedded collagen-laminin scaffolds augment functional recovery after spinal cord injury in mice. Ann N Y Acad Sci. 2020;1465:99–116. 31800108 10.1111/nyas.14279

[138] Baskakov IV, Katorcha E. Multifaceted role of sialylation in prion diseases. Front Neurosci. 2016;10:358. 27551257 10.3389/fnins.2016.00358 PMC4976111

[139] Prusiner SB. Novel proteinaceous infectious particles cause scrapie. Science. 1982;216:136–44. 6801762 10.1126/science.6801762

[140] Borchelt StahlN, Hsiao DR, Prusiner K. SB. Scrapie prion protein contains a phosphatidylinositol glycolipid. Cell. 1987;51:229–40. 2444340 10.1016/0092-8674(87)90150-4

[141] Endo T, Groth D, Prusiner SB, Kobata A. Diversity of oligosaccharide structures linked to asparagines of the scrapie prion protein. Biochemistry. 1989;28:8380–8. 2574992 10.1021/bi00447a017

[142] Stimson E, Hope J, Chong A, Burlingame AL. Site-specific characterization of the N-linked glycans of murine prion protein by high-performance liquid chromatography/electrospray mass spectrometry and exoglycosidase digestions. Biochemistry. 1999;38:4885–95. 10200178 10.1021/bi982330q

[143] Bate C, Nolan W, Williams A. Sialic acid on the glycosylphosphatidylinositol anchor regulates PrP-mediated cell signaling and prion formation. J Biol Chem. 2016;291:160–70. 26553874 10.1074/jbc.M115.672394 PMC4697153

[144] Katorcha E, Makarava N, Savtchenko R, D’Azzo A, Baskakov IV. Sialylation of prion protein controls the rate of prion amplification, the cross-species barrier, the ratio of PrPSc glycoform and prion infectivity. PLoS Pathog. 2014;10:e1004366. 25211026 10.1371/journal.ppat.1004366 PMC4161476

[145] Katorcha E, Srivastava S, Klimova N, Baskakov IV. Sialylation of glycosylphosphatidylinositol (GPI) anchors of mammalian prions is regulated in a host-, tissue-, and cell-specific manner. J Biol Chem. 2016;291:17009–19. 27317661 10.1074/jbc.M116.732040 PMC5016106

[146] Srivastava S, Katorcha E, Daus ML, Lasch P, Beekes M, Baskakov IV. Sialylation controls prion fate in vivo. J Biol Chem. 2017;292:2359–68. 27998976 10.1074/jbc.M116.768010 PMC5313106

[147] Wille H, Michelitsch MD, Guenebaut V, Supattapone S, Serban A, Cohen FE, et al. Structural studies of the scrapie prion protein by electron crystallography. Proc Natl Acad Sci USA. 2002;99:3563–8. 11891310 10.1073/pnas.052703499 PMC122563

[148] Govaerts C, Wille H, Prusiner SB, Cohen FE. Evidence for assembly of prions with left-handed beta-helices into trimers. Proc Natl Acad Sci USA. 2004;101:8342–7. 15155909 10.1073/pnas.0402254101 PMC420396

[149] Requena JR, Wille H. The structure of the infectious prion protein: experimental data and molecular models. Prion. 2014;8:60–6. 24583975 10.4161/pri.28368 PMC7030906

[150] Katorcha E, Makarava N, Savtchenko R, Baskakov IV. Sialylation of the prion protein glycans controls prion replication rate and glycoform ratio. Sci Rep. 2015;5:16912. 26576925 10.1038/srep16912 PMC4649626

[151] Bate C, Williams A. Clustering of sialylated glycosylphosphatidylinositol anchors mediates PrP-induced activation of cytoplasmic phospholipase A 2 and synapse damage. Prion. 2012;6:350–3. 22895089 10.4161/pri.21751 PMC3609062

[152] Bate C, Williams A. Neurodegeneration induced by clustering of sialylated glycosylphosphatidylinositols of prion proteins. J Biol Chem. 2012;287:7935–44. 22262833 10.1074/jbc.M111.275743 PMC3318732

[153] Solomon IH, Khatri N, Biasini E, Massignan T, Huettner JE, Harris DA. An N-terminal polybasic domain and cell surface localization are required for mutant prion protein toxicity. J Biol Chem. 2011;286:14724–36. 21385869 10.1074/jbc.M110.214973 PMC3077669

[154] Westergard L, Turnbaugh JA, Harris DA. A nine amino acid domain is essential for mutant prion protein toxicity. J Neurosci. 2011;31:14005–17. 21957261 10.1523/JNEUROSCI.1243-11.2011 PMC3227396

[155] Keppler OT, Hinderlich S, Langner J, Schwartz-Albiez R, Reutter W, Pawlita M. UDP-GlcNAc 2-epimerase: a regulator of cell surface sialylation. Science. 1999;284:1372–6. 10334995 10.1126/science.284.5418.1372

[156] Guo S, Tian H, Dong R, Yang N, Zhang Y, Yao S, et al. Exogenous supplement of N-acetylneuraminic acid ameliorates atherosclerosis in apolipoprotein E-deficient mice. Atherosclerosis. 2016;251:183–91. 27344369 10.1016/j.atherosclerosis.2016.05.032

[157] Yu L, Peng J, Mineo C. Lipoprotein sialylation in atherosclerosis: lessons from mice. Front Endocrinol. 2022;13:953165. 10.3389/fendo.2022.953165 PMC9498574 36157440

[158] Orekhov AN, Bobryshev YV, Sobenin IA, Melnichenko AA, Chistiakov DA. Modified low density lipoprotein and lipoprotein-containing circulating immune complexes as diagnostic and prognostic biomarkers of atherosclerosis and type 1 diabetes macrovascular disease. Int J Mol Sci. 2014;15:12807–41. 25050779 10.3390/ijms150712807 PMC4139876

[159] Ruelland A, Gallou G, Legras B, Paillard F, Cloarec L. LDL sialic acid content in patients with coronary artery disease. Clin Chim Acta. 1993;221:127–33. 8149630 10.1016/0009-8981(93)90027-2

[160] Tertov VV, Orekhov AN, Sobenin IA, Morrisett JD, Gotto AM Jr, Guevara JG Jr. Carbohydrate composition of protein and lipid components in sialic acid-rich and -poor low density lipoproteins from subjects with and without coronary artery disease. J Lipid Res. 1993;34:365–75. 8468522

[161] Orekhov AN, Tertov VV, Sobenin IA, Smirnov VN, Via DP, Guevara J Jr, et al. Sialic acid content of human low density lipoproteins affects their interaction with cell receptors and intracellular lipid accumulation. J lipid Res. 1992;33:805–17. 1512508

[162] Sukhorukov V, Gudelj I, Pučić-Baković M, Zakiev E, Orekhov A, Kontush A, et al. Glycosylation of human plasma lipoproteins reveals a high level of diversity, which directly impacts their functional properties. Biochim Biophys Acta Mol Cell Biol Lipids. 2019;1864:643–53. 30641224 10.1016/j.bbalip.2019.01.005

[163] Grewal T, Bartlett A, Burgess JW, Packer NH, Stanley KK. Desialylated LDL uptake in human and mouse macrophages can be mediated by a lectin receptor. Atherosclerosis. 1996;121:151–63. 8678920 10.1016/0021-9150(95)05715-3

[164] Tertov VV, Sobenin IA, Gabbasov ZA, Popov EG, Jaakkola O, Solakivi T, et al. Multiple-modified desialylated low density lipoproteins that cause intracellular lipid accumulation. Isolation, fractionation and characterization. Lab Investig. 1992;67:665–75. 1434544

[165] Orekhov AN, Tertov VV, Kabakov AE, Adamova I, Pokrovsky SN, Smirnov VN. Autoantibodies against modified low density lipoprotein. Nonlipid factor of blood plasma that stimulates foam cell formation. Arteriosclerosis and Thrombosis: A Journal of Vascular Biology. 1991;11:316–26. a journal of vascular biology 1998649 10.1161/01.atv.11.2.316

[166] Kacharava AG, Tertov VV, Orekhov AN. Autoantibodies against low-density lipoprotein and atherogenic potential of blood. Ann Med. 1993;25:551–5. 8292305

[167] Tertov VV, Sobenin IA, Orekhov AN, Jaakkola O, Solakivi T, Nikkari T. Characteristics of low density lipoprotein isolated from circulating immune complexes. Atherosclerosis. 1996;122:191–9. 8769682 10.1016/0021-9150(95)05737-4

[168] Fulop T Jr, Larbi A, Fortun A, Robert L, Khalil A. Elastin peptides induced oxidation of LDL by phagocytic cells. Pathol Biol. 2005;53:416–23. 16085119 10.1016/j.patbio.2004.12.023

[169] Robert L, Jacob MP, Frances C, Godeau G, Hornebeck W. Interaction between elastin and elastases and its role in the aging of the arterial wall, skin and other connective tissues. A review. Mech Ageing Dev. 1984;28:155–66. 6394911 10.1016/0047-6374(84)90015-0

[170] Mochizuki S, Brassart B, Hinek A. Signaling pathways transduced through the elastin receptor facilitate proliferation of arterial smooth muscle cells. J Biol Chem. 2002;277:44854–63. 12244048 10.1074/jbc.M205630200

[171] Milner CarrilloMB, Ball CM, Snoek ST, Campbell M. RD. Cloning and characterization of a sialidase from the murine histocompatibility-2 complex: low levels of mRNA and a single amino acid mutation are responsible for reduced sialidase activity in mice carrying the Neu1a allele. Glycobiology. 1997;7:975–86. 9363440 10.1093/glycob/7.7.975

[172] Heimerl M, Sieve I, Ricke-Hoch M, Erschow S, Battmer K, Scherr M, et al. Neuraminidase-1 promotes heart failure after ischemia/reperfusion injury by affecting cardiomyocytes and invading monocytes/macrophages. Basic Res Cardiol. 2020;115:62. 32975669 10.1007/s00395-020-00821-z PMC7519006

[173] Demina EP, Smutova V, Pan X, Fougerat A, Guo T, Zou C, et al. Neuraminidases 1 and 3 trigger atherosclerosis by desialylating low-density lipoproteins and increasing their uptake by macrophages. J Am Heart Assoc. 2021;10:e018756. 33554615 10.1161/JAHA.120.018756 PMC7955353

[174] Hanson VA, Shettigar UR, Loungani RR, Nadijcka MD. Plasma sialidase activity in acute myocardial infarction. Am Heart J. 1987;114:59–63. 3604873 10.1016/0002-8703(87)90307-3

[175] Gracheva EV, Samovilova NN, Golovanova NK, Il’inskaya OP, Tararak EM, Malyshev PP, et al. Sialyltransferase activity of human plasma and aortic intima is enhanced in atherosclerosis. Biochim Biophys Acta. 2002;1586:123–8. 11781157 10.1016/s0925-4439(01)00093-x

[176] Döring Y, Noels H, Mandl M, Kramp B, Neideck C, Lievens D, et al. Deficiency of the sialyltransferase St3Gal4 reduces Ccl5-mediated myeloid cell recruitment and arrest: short communication. Circ Res. 2014;114:976–81. 24425712 10.1161/CIRCRESAHA.114.302426 PMC4353583

[177] Saade S, Cazier JB, Ghassibe-Sabbagh M, Youhanna S, Badro DA, Kamatani Y, et al. Large scale association analysis identifies three susceptibility loci for coronary artery disease. PloS ONE. 2011;6:e29427. 22216278 10.1371/journal.pone.0029427 PMC3246490

[178] Zhang J, Liu Y, Deng X, Chen L, Yang X, Yu C. ST6GAL1 negatively regulates monocyte transendothelial migration and atherosclerosis development. Biochem Biophys Res Commun. 2018;500:249–55. 29654763 10.1016/j.bbrc.2018.04.053

[179] Stevens J, Blixt O, Glaser L, Taubenberger JK, Palese P, Paulson JC, et al. Glycan microarray analysis of the hemagglutinins from modern and pandemic influenza viruses reveals different receptor specificities. J Mol Biol. 2006;355:1143–55. 16343533 10.1016/j.jmb.2005.11.002

[180] Steele H, Tague AJ, Skropeta D. The role of sialylation in respiratory viral infection and treatment. Curr Med Chem. 2021;28:5251–67. 33593248 10.2174/0929867328666210201153901

[181] Sauter NK, Bednarski MD, Wurzburg BA, Hanson JE, Whitesides GM, Skehel JJ, et al. Hemagglutinins from two influenza virus variants bind to sialic acid derivatives with millimolar dissociation constants: a 500-MHz proton nuclear magnetic resonance study. Biochemistry. 1989;28:8388–96. 2605190 10.1021/bi00447a018

[182] Neu U, Bauer J, Stehle T. Viruses and sialic acids: rules of engagement. Curr Opin Struct Biol. 2011;21:610–8. 21917445 10.1016/j.sbi.2011.08.009 PMC3189341

[183] Gamblin SJ, Skehel JJ. Influenza hemagglutinin and neuraminidase membrane glycoproteins. J Biol Chem. 2010;285:28403–9. 20538598 10.1074/jbc.R110.129809 PMC2937864

[184] Wagner R, Matrosovich M, Klenk HD. Functional balance between haemagglutinin and neuraminidase in influenza virus infections. Rev Med Virol. 2002;12:159–66. 11987141 10.1002/rmv.352

[185] Baum LG, Paulson JC. The N2 neuraminidase of human influenza virus has acquired a substrate specificity complementary to the hemagglutinin receptor specificity. Virology. 1991;180:10–5. 1984642 10.1016/0042-6822(91)90003-t

[186] Zeng Q, Langereis MA, van Vliet AL, Huizinga EG, de Groot RJ. Structure of coronavirus hemagglutinin-esterase offers insight into corona and influenza virus evolution. Proc Natl Acad Sci USA. 2008;105:9065–9. 18550812 10.1073/pnas.0800502105 PMC2449365

[187] Arai M, Yamada K, Toyota T, Obata N, Haga S, Yoshida Y, et al. Association between polymorphisms in the promoter region of the sialyltransferase 8B (SIAT8B) gene and schizophrenia. Biol psychiatry. 2006;59:652–9. 16229822 10.1016/j.biopsych.2005.08.016

[188] Isomura R, Kitajima K, Sato C. Structural and functional impairments of polysialic acid by a mutated polysialyltransferase found in schizophrenia. J Biol Chem. 2011;286:21535–45. 21464126 10.1074/jbc.M111.221143 PMC3122212

[189] Anney R, Klei L, Pinto D, Regan R, Conroy J, Magalhaes TR, et al. A genome-wide scan for common alleles affecting risk for autism. Hum Mol Genet. 2010;19:4072–82. 20663923 10.1093/hmg/ddq307 PMC2947401

[190] Kamien B, Harraway J, Lundie B, Smallhorne L, Gibbs V, Heath A, et al. Characterization of a 520 kb deletion on chromosome 15q26.1 including ST8SIA2 in a patient with behavioral disturbance, autism spectrum disorder, and epilepsy: additional information. Am J Med Genet Part A. 2015;167:1424. 25846131 10.1002/ajmg.a.36846

[191] Barbeau D, Liang JJ, Robitalille Y, Quirion R, Srivastava LK. Decreased expression of the embryonic form of the neural cell adhesion molecule in schizophrenic brains. Proc Natl Acad Sci USA. 1995;92:2785–9. 7708724 10.1073/pnas.92.7.2785 PMC42303

[192] Gilabert-Juan J, Varea E, Guirado R, Blasco-Ibáñez JM, Crespo C, Nácher J. Alterations in the expression of PSA-NCAM and synaptic proteins in the dorsolateral prefrontal cortex of psychiatric disorder patients. Neurosci Lett. 2012;530:97–102. 23022470 10.1016/j.neulet.2012.09.032

[193] Varea E, Guirado R, Gilabert-Juan J, Martí U, Castillo-Gomez E, Blasco-Ibáñez JM, et al. Expression of PSA-NCAM and synaptic proteins in the amygdala of psychiatric disorder patients. J Psychiatr Res. 2012;46:189–97. 22099865 10.1016/j.jpsychires.2011.10.011

[194] Kröcher T, Malinovskaja K, Jürgenson M, Aonurm-Helm A, Zharkovskaya T, Kalda A, et al. Schizophrenia-like phenotype of polysialyltransferase ST8SIA2-deficient mice. Brain Struct Funct. 2015;220:71–83. 24057454 10.1007/s00429-013-0638-z

[195] Bowles WHD, Gloster TM. Sialidase and sialyltransferase inhibitors: targeting pathogenicity and disease. Front Mol Biosci. 2021;8:705133. 34395532 10.3389/fmolb.2021.705133 PMC8358268

[196] Wang L, Liu Y, Wu L, Sun XL. Sialyltransferase inhibition and recent advances. Biochim Biophys Acta. 2016;1864:143–53. 26192491 10.1016/j.bbapap.2015.07.007

[197] Moons SJ, Rossing E, Janssen M, Heise T, Büll C, Adema GJ, et al. Structure-activity relationship of metabolic sialic acid inhibitors and labeling reagents. ACS Chem Biol. 2022;17:590–7. 35179348 10.1021/acschembio.1c00868 PMC8938927

[198] Büll C, Boltje TJ, Wassink M, de Graaf AM, van Delft FL, den Brok MH, et al. Targeting aberrant sialylation in cancer cells using a fluorinated sialic acid analog impairs adhesion, migration, and in vivo tumor growth. Mol Cancer Ther. 2013;12:1935–46. 23974695 10.1158/1535-7163.MCT-13-0279

[199] Büll C, Boltje TJ, van Dinther EA, Peters T, de Graaf AM, Leusen JH, et al. Targeted delivery of a sialic acid-blocking glycomimetic to cancer cells inhibits metastatic spread. ACS Nano. 2015;9:733–45. 25575241 10.1021/nn5061964

[200] Büll C, Boltje TJ, Balneger N, Weischer SM, Wassink M, van Gemst JJ, et al. Sialic acid blockade suppresses tumor growth by enhancing T-cell-mediated tumor immunity. Cancer Res. 2018;78:3574–88. 29703719 10.1158/0008-5472.CAN-17-3376

[201] Heise T, Pijnenborg JFA, Büll C, van Hilten N, Kers-Rebel ED, Balneger N, et al. Potent metabolic sialylation inhibitors based on C-5-modified fluorinated sialic acids. J Med Chem. 2019;62:1014–21. 30543426 10.1021/acs.jmedchem.8b01757 PMC6348442

[202] Macauley MS, Arlian BM, Rillahan CD, Pang PC, Bortell N, Marcondes MC, et al. Systemic blockade of sialylation in mice with a global inhibitor of sialyltransferases. J Biol Chem. 2014;289:35149–58. 25368325 10.1074/jbc.M114.606517 PMC4271204

[203] Hsu CC, Lin TW, Chang WW, Wu CY, Lo WH, Wang PH, et al. Soyasaponin-I-modified invasive behavior of cancer by changing cell surface sialic acids. Gynecol Oncol. 2005;96:415–22. 15661230 10.1016/j.ygyno.2004.10.010

[204] Hunter C, Gao Z, Chen HM, Thompson N, Wakarchuk W, Nitz M, et al. Attenuation of polysialic acid biosynthesis in cells by the small molecule inhibitor 8-keto-sialic acid. ACS Chem Biol. 2023;18:41–8. 36577399 10.1021/acschembio.2c00638

[205] Ahuja S, Cahill J, Hartfield K, Whorton MR. Inhibition of CMP-sialic acid transport by endogenous 5-methyl CMP. PloS ONE. 2021;16:e0249905. 34081697 10.1371/journal.pone.0249905 PMC8174729

[206] Muz B, Abdelghafer A, Markovic M, Yavner J, Melam A, Salama NN, et al. Targeting E-selectin to tackle cancer using uproleselan. Cancers. 2021;13:335. 33477563 10.3390/cancers13020335 PMC7831123

[207] Ho CH, Chen ML, Huang HL, Lai CJ, Liu CH, Chuu CP, et al. Active targeting of P-selectin by fucoidan modulates the molecular profiling of metastasis in docetaxel-resistant prostate cancer. Mar drugs. 2022;20:542. 36135731 10.3390/md20090542 PMC9500773

[208] Cai Z, Yan Y, Zhou J, Yang Y, Zhang Y, Chen J. Multifunctionalized brush-like glycopolymers with high affinity to p-selectin and antitumor metastasis activity. Biomacromolecules. 2021;22:1177–85. 33586430 10.1021/acs.biomac.0c01689

[209] Manni M, Läubli H. Targeting glyco-immune checkpoints for cancer therapy. Expert Opin Biol Ther. 2021;21:1063–71. 33502268 10.1080/14712598.2021.1882989

[210] Bartish M, Del Rincón SV, Rudd CE, Saragovi HU. Aiming for the sweet spot: glyco-immune checkpoints and γδ T cells in targeted immunotherapy. Front Immunol. 2020;11:564499. 33133075 10.3389/fimmu.2020.564499 PMC7550643

[211] Gray MA, Stanczak MA, Mantuano NR, Xiao H, Pijnenborg JFA, Malaker SA, et al. Targeted glycan degradation potentiates the anticancer immune response in vivo. Nat Chem Biol. 2020;16:1376–84. 32807964 10.1038/s41589-020-0622-x PMC7727925

[212] Bärenwaldt A, Läubli H. The sialoglycan-Siglec glyco-immune checkpoint—a target for improving innate and adaptive anti-cancer immunity. Expert Opin Ther Targets. 2019;23:839–53. 31524529 10.1080/14728222.2019.1667977

[213] Ibarlucea-Benitez I, Weitzenfeld P, Smith P, Ravetch JV. Siglecs-7/9 function as inhibitory immune checkpoints in vivo and can be targeted to enhance therapeutic antitumor immunity. Proc Natl Acad Sci USA. 2021;118:e2107424118. 34155121 10.1073/pnas.2107424118 PMC8256000

[214] Haas Q, Boligan KF, Jandus C, Schneider C, Simillion C, Stanczak MA, et al. Siglec-9 regulates an effector memory CD8(+) T-cell subset that congregates in the melanoma tumor microenvironment. Cancer Immunol Res. 2019;7:707–18. 30988027 10.1158/2326-6066.CIR-18-0505

[215] Choi H, Ho M, Adeniji OS, Giron L, Bordoloi D, Kulkarni AJ, et al. Development of Siglec-9 blocking antibody to enhance anti-tumor immunity. Front Oncol. 2021;11:778989. 34869028 10.3389/fonc.2021.778989 PMC8640189

[216] Wang J, Sun J, Liu LN, Flies DB, Nie X, Toki M, et al. Siglec-15 as an immune suppressor and potential target for normalization cancer immunotherapy. Nat Med. 2019;25:656–66. 30833750 10.1038/s41591-019-0374-x PMC7175920

[217] He F, Wang N, Li J, He L, Yang Z, Lu J, et al. High affinity monoclonal antibody targeting Siglec-15 for cancer immunotherapy. J Clin Transl Res. 2021;7:739–49. 34988324 PMC8710358

[218] Zhou ZR, Wang XY, Jiang L, Li DW, Qian RC. Sialidase-conjugated “NanoNiche” for efficient immune checkpoint blockade therapy. ACS Appl bio Mater. 2021;4:5735–41. 35006749 10.1021/acsabm.1c00507

[219] Sharma M, Lathers D, Johnson M, Luke J, Puzanov I, Curti B, et al. 772 A phase 1/2 dose escalation/expansion study evaluating the safety, pharmacokinetics, pharmacodynamics, and antitumor activity of E-602, a bi-sialidase fusion protein, in advanced cancer (GLIMMER-01). J Immunother Cancer. 2022;10. 10.1136/jitc-2022-SITC2022.0772.

[220] Yu S, Wang Q, Zhang J, Wu Q, Guo Z. Synthesis and evaluation of protein conjugates of GM3 derivatives carrying modified sialic acids as highly immunogenic cancer vaccine candidates. MedChemComm. 2011;2:524–30. 21927709 10.1039/C1MD00033K PMC3172705

[221] Wu J, Guo Z. Improving the antigenicity of sTn antigen by modification of its sialic acid residue for development of glycoconjugate cancer vaccines. Bioconjug Chem. 2006;17:1537–44. 17105234 10.1021/bc060103s PMC2532825

[222] Miles D, Papazisis K. Rationale for the clinical development of STn-KLH (Theratope) and anti-MUC-1 vaccines in breast cancer. Clin Breast Cancer. 2003;3:S134–8. 12620150 10.3816/cbc.2003.s.002

[223] O’Cearbhaill RE, Ragupathi G, Zhu J, Wan Q, Mironov S, Yang G, et al. A phase I study of unimolecular pentavalent (Globo-H-GM2-sTn-TF-Tn) immunization of patients with epithelial ovarian, fallopian tube, or peritoneal cancer in first remission. Cancers. 2016;8:46. 27110823 10.3390/cancers8040046 PMC4846855

[224] Sabbatini PJ, Ragupathi G, Hood C, Aghajanian CA, Juretzka M, Iasonos A, et al. Pilot study of a heptavalent vaccine-keyhole limpet hemocyanin conjugate plus QS21 in patients with epithelial ovarian, fallopian tube, or peritoneal cancer. Clin Cancer Res. 2007;13:4170–7. 17634545 10.1158/1078-0432.CCR-06-2949

[225] Paul S, Candelario-Jalil E. Emerging neuroprotective strategies for the treatment of ischemic stroke: An overview of clinical and preclinical studies. Exp Neurol. 2021;335:113518. 33144066 10.1016/j.expneurol.2020.113518 PMC7869696

[226] Jayaraj RL, Azimullah S, Beiram R, Jalal FY, Rosenberg GA. Neuroinflammation: friend and foe for ischemic stroke. J Neuroinflammation. 2019;16:142. 31291966 10.1186/s12974-019-1516-2 PMC6617684

[227] Amani H, Habibey R, Shokri F, Hajmiresmail SJ, Akhavan O, Mashaghi A, et al. Selenium nanoparticles for targeted stroke therapy through modulation of inflammatory and metabolic signaling. Sci Rep. 2019;9:6044. 30988361 10.1038/s41598-019-42633-9 PMC6465364

[228] Xue Y, Dongmei L, Yige Z, Hang G, Li H. Angelica polysaccharide moderates hypoxia-evoked apoptosis and autophagy in rat neural stem cells by downregulation of BNIP3. Artif Cells Nanomed Biotechnol. 2019;47:2492–9. 31208217 10.1080/21691401.2019.1623228

[229] Wielgat P, Niemirowicz-Laskowska K, Wilczewska AZ, Car H. Sialic acid-modified nanoparticles-new approaches in the glioma management-perspective review. Int J Mol Sci. 2021;22:7494. 34299113 10.3390/ijms22147494 PMC8304714

[230] Kuo YC, Wang LJ, Rajesh R. Targeting human brain cancer stem cells by curcumin-loaded nanoparticles grafted with anti-aldehyde dehydrogenase and sialic acid: Colocalization of ALDH and CD44. Mater Sci Eng C Mater Biol Appl. 2019;102:362–72. 31147008 10.1016/j.msec.2019.04.065

[231] Su Y, Guo C, Chen Q, Guo H, Wang J, Kaihang M, et al. Novel multifunctional bionanoparticles modified with sialic acid for stroke treatment. Int J Biol Macromol. 2022;214:278–89. 35716787 10.1016/j.ijbiomac.2022.06.102

[232] Toots M, Plemper RK. Next-generation direct-acting influenza therapeutics. Transl Res J Lab Clin Med. 2020;220:33–42. 10.1016/j.trsl.2020.01.005 PMC7102518 32088166

[233] Matsubara T, Onishi A, Saito T, Shimada A, Inoue H, Taki T, et al. Sialic acid-mimic peptides as hemagglutinin inhibitors for anti-influenza therapy. J Med Chem. 2010;53:4441–9. 20476787 10.1021/jm1002183

[234] Gambaryan AS, Tuzikov AB, Chinarev AA, Juneja LR, Bovin NV, Matrosovich MN. Polymeric inhibitor of influenza virus attachment protects mice from experimental influenza infection. Antivir Res. 2002;55:201–5. 12076764 10.1016/s0166-3542(02)00020-7

[235] Hendricks GL, Weirich KL, Viswanathan K, Li J, Shriver ZH, Ashour J, et al. Sialylneolacto-N-tetraose c (LSTc)-bearing liposomal decoys capture influenza A virus. J Biol Chem. 2013;288:8061–73. 23362274 10.1074/jbc.M112.437202 PMC3605625

[236] Russell RJ, Stevens DJ, Haire LF, Gamblin SJ, Skehel JJ. Avian and human receptor binding by hemagglutinins of influenza A viruses. Glycoconj J. 2006;23:85–92. 16575525 10.1007/s10719-006-5440-1

[237] Koszalka P, Tilmanis D, Hurt AC. Influenza antivirals currently in late-phase clinical trial. Influenza Other Respir. Viruses. 2017;11:240–6. 28146320 10.1111/irv.12446 PMC5410715

[238] Malakhov MP, Aschenbrenner LM, Smee DF, Wandersee MK, Sidwell RW, Gubareva LV, et al. Sialidase fusion protein as a novel broad-spectrum inhibitor of influenza virus infection. Antimicrob Agents Chemother. 2006;50:1470–9. 16569867 10.1128/AAC.50.4.1470-1479.2006 PMC1426979

[239] Zenilman JM, Fuchs EJ, Hendrix CW, Radebaugh C, Jurao R, Nayak SU, et al. Phase 1 clinical trials of DAS181, an inhaled sialidase, in healthy adults. Antivir Res. 2015;123:114–9. 26391974 10.1016/j.antiviral.2015.09.008 PMC4639451

[240] Mascarenhas JX, Korokhov N, Burger L, Kassim A, Tuter J, Miller D, et al. Genetic engineering of CHO cells for viral resistance to minute virus of mice. Biotechnol Bioeng. 2017;114:576–88. 27642072 10.1002/bit.26186

[241] Wu D, Huang W, Wang Y, Guan W, Li R, Yang Z, et al. Gene silencing of β-galactosamide α-2,6-sialyltransferase 1 inhibits human influenza virus infection of airway epithelial cells. BMC Microbiol. 2014;14:78. 24670114 10.1186/1471-2180-14-78 PMC3986885

[242] Szabo R, Skropeta D. Advancement of sialyltransferase inhibitors: therapeutic challenges and opportunities. Med Res Rev. 2017;37:219–70. 27678392 10.1002/med.21407

[243] Glaser L, Conenello G, Paulson J, Palese P. Effective replication of human influenza viruses in mice lacking a major alpha2,6 sialyltransferase. Virus Res. 2007;126:9–18. 17313986 10.1016/j.virusres.2007.01.011

[244] Devaux CA, Rolain JM, Colson P, Raoult D. New insights on the antiviral effects of chloroquine against coronavirus: what to expect for COVID-19? Int J Antimicrob Agents. 2020;55:105938. 32171740 10.1016/j.ijantimicag.2020.105938 PMC7118659

[245] Varki A. Sialic acids as ligands in recognition phenomena. FASEB J. 1997;11:248–55. 9068613 10.1096/fasebj.11.4.9068613

[246] Fantini J, Di Scala C, Chahinian H, Yahi N. Structural and molecular modelling studies reveal a new mechanism of action of chloroquine and hydroxychloroquine against SARS-CoV-2 infection. Int J Antimicrob Agents. 2020;55:105960. 32251731 10.1016/j.ijantimicag.2020.105960 PMC7128678

[247] Linnartz B, Wang Y, Neumann H. Microglial immunoreceptor tyrosine-based activation and inhibition motif signaling in neuroinflammation. Int J Alzheimer’s Dis. 2010;2010:587463. 20721346 10.4061/2010/587463 PMC2915791

[248] Wang Y, Neumann H. Alleviation of neurotoxicity by microglial human Siglec-11. J Neurosci. 2010;30:3482–8. 20203208 10.1523/JNEUROSCI.3940-09.2010 PMC6634112

[249] Shahraz A, Kopatz J, Mathy R, Kappler J, Winter D, Kapoor S, et al. Anti-inflammatory activity of low molecular weight polysialic acid on human macrophages. Sci Rep. 2015;5:16800. 26582367 10.1038/srep16800 PMC4652165

[250] Bondioli L, Ruozi B, Belletti D, Forni F, Vandelli MA, Tosi G. Sialic acid as a potential approach for the protection and targeting of nanocarriers. Expert Opin Drug Deliv. 2011;8:921–37. 21510826 10.1517/17425247.2011.577061

[251] Gabizon A, Papahadjopoulos D. Liposome formulations with prolonged circulation time in blood and enhanced uptake by tumors. Proc Natl Acad Sci USA. 1988;85:6949–53. 3413128 10.1073/pnas.85.18.6949 PMC282096

[252] Liu D, Mori A, Huang L. Large liposomes containing ganglioside GM1 accumulate effectively in spleen. Biochim Biophys Acta. 1991;1066:159–65. 1854781 10.1016/0005-2736(91)90182-8

[253] Liu D, Mori A, Huang L. Role of liposome size and RES blockade in controlling biodistribution and tumor uptake of GM1-containing liposomes. Biochim Biophys Acta. 1992;1104:95–101. 1550858 10.1016/0005-2736(92)90136-a

[254] Taira MC, Chiaramoni NS, Pecuch KM, Alonso-Romanowski S. Stability of liposomal formulations in physiological conditions for oral drug delivery. Drug Deliv. 2004;11:123–8. 15200011 10.1080/10717540490280769

