# Supplementary material for: Biological function of sialic acid and sialylation in human health and disease
Source: Cell Death Discov. 2024 Sep 30;10:415. doi: 10.1038/s41420-024-02180-3 (PMC11442784; doi:10.1038/s41420-024-02180-3)
Supplement: Supplementary file 1 — Agents Targeting Sialylation for Disease Treatment [file 41420_2024_2180_MOESM1_ESM.docx]

**Supplementary Table: Agents Targeting Sialylation for Disease Treatment**

| **Disease/Application** | **Specific Agent** | **Agent Type** | **Target/Mechanism** | **Stage of Development** |
| --- | --- | --- | --- | --- |
| **Anti-cancer** | P-3Fax-Neu5Ac | Mimic | Abolishing SLex expression and reducing E-selectin and P-selectin binding.  Enhancing cytotoxic CD8+ T cell-mediated anti-tumour response. | Animal trials (mice) |
|  | Soyasaponin I | Inhibitor | Suppressing cellular ST3Gal activity, inducing the decrease of tumor cell invasiveness | Animal trials (mice) |
|  | 8-Keto-sialic acid | Inhibitor | Terminating the elongation of α2,8-linked sialic acid chains and endogenous CMP-Neu5Ac, suppressing tumour metastasis | Cell experiments |
|  | Uproleselan | Mimic | Affecting tumour cell extravasation and adhesion via blocking E-selectin, limiting tumour metastasis | Phase Ⅰ/Ⅱ Clinical trials |
|  | Fucoidan | Adjuvant | Combined with chemotherapy, targeting P-selectin to limit tumour metastasis. | Clinical trial |
|  | E-602 | Blocker | Blocking the binding of sialic acids to SIGLECs, inhibiting tumour cell hypersialylation-mediated immune evasion | Phase Ⅰ/Ⅱ Clinical trials |
|  | STn-KLH vaccine | Adjuvant and Vaccine | Improving sTn antigenicity, eliciting high titres of antigen-specific IgG antibodies | Clinical trials completed |
|  | Globo-H–GM2–sTn–TF–Tn vaccine | Adjuvant and Vaccine | Improving sTn antigenicity, eliciting high titres of antigen-specific IgG antibodies | Phase Ⅰ Clinical trial |
|  | MUC1 vaccine | Adjuvants and Vaccine | Improving sTn antigenicity, eliciting high titres of antigen-specific IgG antibodies | Phase Ⅰ/Ⅱ Clinical trial |
| **Anti-respiratory virus** | Oseltamivir | Inhibitor | Competitively inhibiting NA from hydrolysing the bonds between HA and the terminal sialic acids from the host sialoglycan chain | Clinical application |
|  | Zanamivir | Inhibitor | Competitively inhibiting NA from hydrolysing the bonds between HA and the terminal sialic acids from the host sialoglycan chain | Clinical application |
|  | Peptide Ala-Arg-Leu-Pro-Arg | Mimic | Sialic acid analogue, blocking HA-mediated binding of virus to the sialylgalactose linkages of host cell receptors. | Cell experiments |
|  | Sialylneolacto-N-tetraose c | Mimic | Sialic acid analogue, blocking HA-mediated binding of virus to the sialylgalactose linkages of host cell receptors. | Cell and animal trials (mice) |
|  | DAS-181 (Fludase) | Mimic | Sialidase mimic, cleaving sialic acids from the host cell surface to prevent the host cell receptors recognized by virus. | Phase III clinical trial |
|  | Chloroquine | Blocker | Binding to sialic acids and sialoglycans, blocking the bonds between the N-terminal domain of the S glycoprotein and sialoglycans, preventing virus attachment. | Clinical application |
| **CNS injury** | Tegaserod | Mimic | Mimicking polySia to promote axonal regrowth across the lesion and neural connections | Animal trials |
|  | 5-nonyloxytryptamine oxalate | Mimic | Mimicking polySia to promote axonal regrowth across the lesion and neural connections | Animal trials |
